# Research on alcohol and other drug (AOD) use among sexual minority women: A global scoping review

**DOI:** 10.1371/journal.pone.0229869

**Published:** 2020-03-18

**Authors:** Tonda L. Hughes, Cindy B. Veldhuis, Laurie A. Drabble, Sharon C. Wilsnack

**Affiliations:** 1 School of Nursing, Columbia University, New York, New York, United States of America; 2 San Jose State University, San Jose, California, United States of America; 3 University of North Dakota, Grand Forks, North Dakota, United States of America; Anglia Ruskin University, UNITED KINGDOM

## Abstract

Until the 1980s, the limited research on alcohol and other drug (AOD) use among sexual minority women (SMW) focused on alcohol and used samples recruited from gay bars, resulting in inflated estimates of hazardous drinking. Over the past several decades the number of AOD studies with SMW has increased dramatically. To characterize this literature, we conducted a scoping review to answer the following questions: *What do we know*, *and what are the gaps in research about AOD use among SMW*? We searched multiple electronic databases (Medline [PubMed], CINAHL, PsycInfo, and Web of Science) for peer-reviewed research articles about AOD use among adult SMW published between January 1, 2000 and May 31, 2017. After duplicates were removed the search identified 4,204 articles. We reviewed the titles and abstracts and removed articles that did not meet inclusion criteria. We used full-text review of the remaining 229 articles to make a final determination regarding inclusion and we retained 181 articles for review. Although the quantity of AOD research with SMW has grown substantially, the great majority of studies have been conducted in the United States (US) and most focus on hazardous drinking; relatively little research has focused on other drugs. In addition, although there has been marked improvement in theories and methods used in this research, many gaps and limitations remain. Examples are the lack of longitudinal research; reliance on samples that tend to over-represent white, well-educated, and relatively young women; sparse attention to mechanisms underlying the disproportionately high rates of AOD use among SMW; and the absence of intervention research. In general, more high-quality research on SMW’s use of AODs is needed, but gaps and limitations are particularly large in non-western countries. Addressing these research gaps and limitations is essential for providing information that can be used to develop more effective prevention and early intervention strategies, as well as for informing policies that can help to reduce risky drinking and drug misuse among SMW.

## Introduction

Over the past two decades an increasing number of studies have reported sexual-orientation-related disparities in behavioral health, mental health, and physical health among lesbian, gay, bisexual, and transgender (LGBT) people. This has resulted in greater understanding of the health care needs of these population groups and the challenges they face in attaining/maintaining good health and in accessing care. However, many gaps in knowledge remain, particularly related to sexual minority women’s health.

Although sexual minority women (SMW; e.g., lesbian or bisexual women, women who have sex with women or who identify as something other than heterosexual) have many of the same health concerns as heterosexual women, substantial sexual-orientation-related health disparities have been reported among women in the US and in other parts of the world [1–8]. Such disparities may be compounded among sexual minorities who are also members of another marginalized group (e.g., racial/ethnic or religious minorities, immigrants), are younger or older persons, have co-occurring health concerns, or live in countries or regions where homosexuality is highly stigmatized or criminalized. Despite the recent growth in research, there have been few attempts to summarize what is currently known about SMW’s health, especially regarding their use of alcohol and other drugs (AODs) [9–12].

Hazardous drinking (HD) is among the most prominent sexual-orientation-related health disparities among women [13]. SMW are much more likely than heterosexual women to drink, drink heavily, and experience alcohol-related problems and alcohol-use disorders [14–17]. For example, they are eleven times as likely as heterosexual women to meet criteria for alcohol dependence, and eight times as likely to report seeking help for alcohol-related problems [18], indicating a disproportionately high risk of health conditions such as injury, liver, and heart diseases [19,20]. Although less research has focused on SMW’s use of drugs other than alcohol, they appear to also be at higher risk than heterosexual women for drug misuse and drug use disorders [21–23]. Use of other drugs can have a wide range of health effects on the body and brain. For example, marijuana use has been associated with certain cancers (i.e., lung, head, and neck), mental health disorders (e.g., anxiety and depression), neurocognitive effects, and injuries [24–27]. The overarching aim of this scoping review was to review existing research on alcohol and other drug use among SMW to characterize gaps and limitations and to make recommendations about how to address them.

## Scoping review methodology

Scoping studies have grown in popularity over the past 10 years and are now commonly used, especially to review health research. Researchers conduct scoping reviews to examine the extent and nature of research activity about a particular topic, and to summarize and disseminate research findings or identify gaps in the existing literature. Scoping reviews are particularly useful in understanding emerging health topics, such as the health concerns of SMW. Tricco and colleagues [28] define a scoping review as a systematic approach to “charting” or “mapping” a broader question than is typically addressed in a systematic review. They are comprehensive reviews that generally do not evaluate the quality of individual studies but focus on identifying gaps in the literature and setting this within research, practice and/or policy arenas. Scoping studies are ideal for moving forward research on AOD use among SMW with the ultimate goal of improving care and reducing sexual-orientation-related health disparities.

### Methods

We used the methodological framework for scoping reviews recommended by Arksey and O’Malley [29] with enhancements by Levac, Colquhoun, and O’Brian [30]. This included five steps: (1) identifying the research question, (2) identifying relevant studies, (3) selecting studies, (4) mapping/charting the data, and (5) collating, summarizing, and reporting the results.

#### Identifying the research question

This scoping review was guided by the research question: *What do we know*, *and what are the gaps in research about AOD use among SMW*?

#### Identifying relevant studies

We developed the search strategy in consultation with an experienced Information Scientist at Columbia University. We searched a range of electronic databases including Medline (PubMed), CINAHL, PsycInfo, and Web of Science (WoS) for articles published from January 1, 2000 through May 2017. Following is an example of the search terms (these terms were used in the PubMed search).

("sexual minority" OR "sexual minorities" OR lesbian OR lesbians OR bisexual OR bisexuals OR bisexuality OR queer OR LGBT OR LGB OR LGBTQ OR LGBTQIA OR "homosexual female" OR "homosexual females" OR "homosexual women" OR "homosexual woman" OR "female homosexuality" OR “same-sex relationships” OR “same-sex female relationships” OR “mostly heterosexual women”) AND (alcohol OR alcoholic OR alcoholism OR “alcohol use disorder” OR "substance use" OR "substance abuse" OR "binge drinking" OR "binge drinker" OR "binge drinkers" OR "binge drink" OR "alcohol consumption" OR "consume alcohol" OR ethanol OR "drug dependence" OR "drug dependency" OR "drug dependent" OR addict OR addicts OR addicted OR addiction OR "drug abuse" OR "drug use disorder" OR tobacco OR cigarette OR nicotine OR e-cigarette OR vape OR vaping OR cigarettes OR smoking OR amphetamine OR methamphetamine OR cocaine OR opioid OR opioids OR heroin OR morphine OR "drug overdose" OR cannabis OR marijuana OR benzo OR fentanyl)

#### Selecting studies

Articles retrieved in the searches were evaluated for fit using the following selection criteria: quantitative studies published in English that reported data about AODs collected from women who identified as lesbian, bisexual or other non-heterosexual identity, or who reported having had a same-sex partner. Given the large number of studies retrieved, and because a recent scoping review [31] focused on substance use among young sexual and gender minorities, we narrowed our search to include only studies of adult (≥ 18-year-old) SMW; we also excluded articles that focused solely on college students. Since 2003, at least seven reviews of cigarette smoking or other tobacco use among sexual minority people have been published. Therefore, we excluded studies that focused solely on nicotine and tobacco use. In addition, because a large proportion of research about sexual minority health, particularly substance use-related research, focuses on HIV/AIDS and other sexually transmitted infections (STIs) [32,33], we excluded studies on these topics. Although we included studies that reflected SMW’s need for treatment, we excluded studies of treatment outcomes or barriers to treatment. We excluded studies of transgender women unless researchers also assessed sexual identity. To ensure rigor, we used an iterative team approach in selecting studies and extracting data.

The selection process included multiple screenings. All references were imported into Endnote and duplicates removed, leaving 4,204 references. Titles were screened to exclude articles that did not fit our inclusion criteria (e.g., articles that reported results of qualitative studies or that focused on men, youth, HIV/AIDS, or transgender individuals). Abstracts of the remaining 371 articles were divided among authors and each abstract reviewed by at least two authors to determine relevance; any differences in determinations about relevance were discussed until agreement was reached. This process resulted in 229 articles. Abstracts about which the authors were uncertain underwent a full-text review. If an article was excluded during the full review phase, we recorded the reason for its exclusion. After full-text reviews were completed 181 articles remained and were included in our review.

#### Mapping/Charting the data

**Fig 1:**
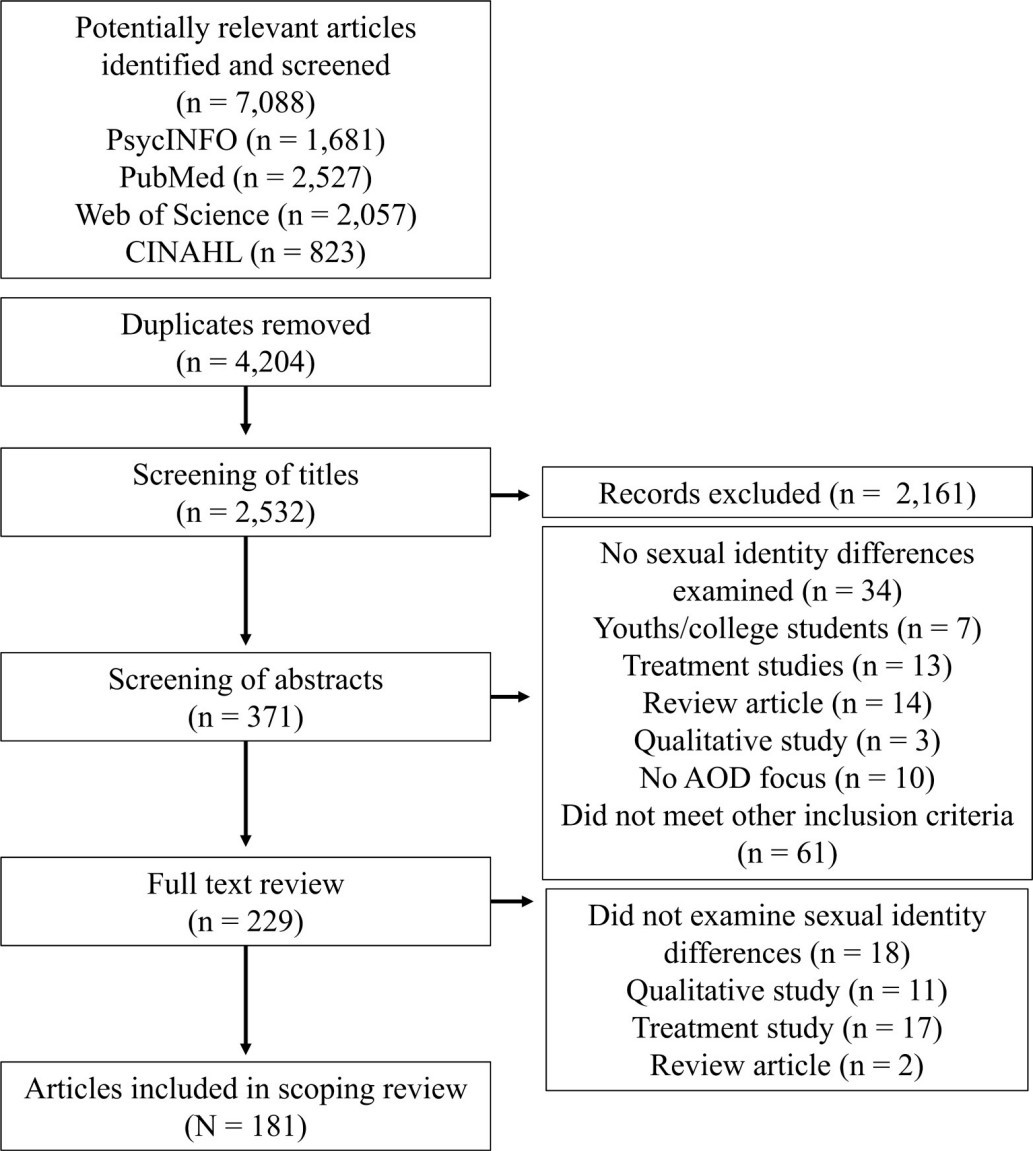
PRISMA flow diagram for scoping reviews showing literature search and selection of articles.

Articles that met inclusion criteria were read and information about the study population(s), study location (country and state/province/region), recruitment method, research question, methods and measures used, and key findings were extracted from each article. Fig 1 summarizes the selection process.

#### Collating, summarizing, and reporting the results

**Fig 2:**
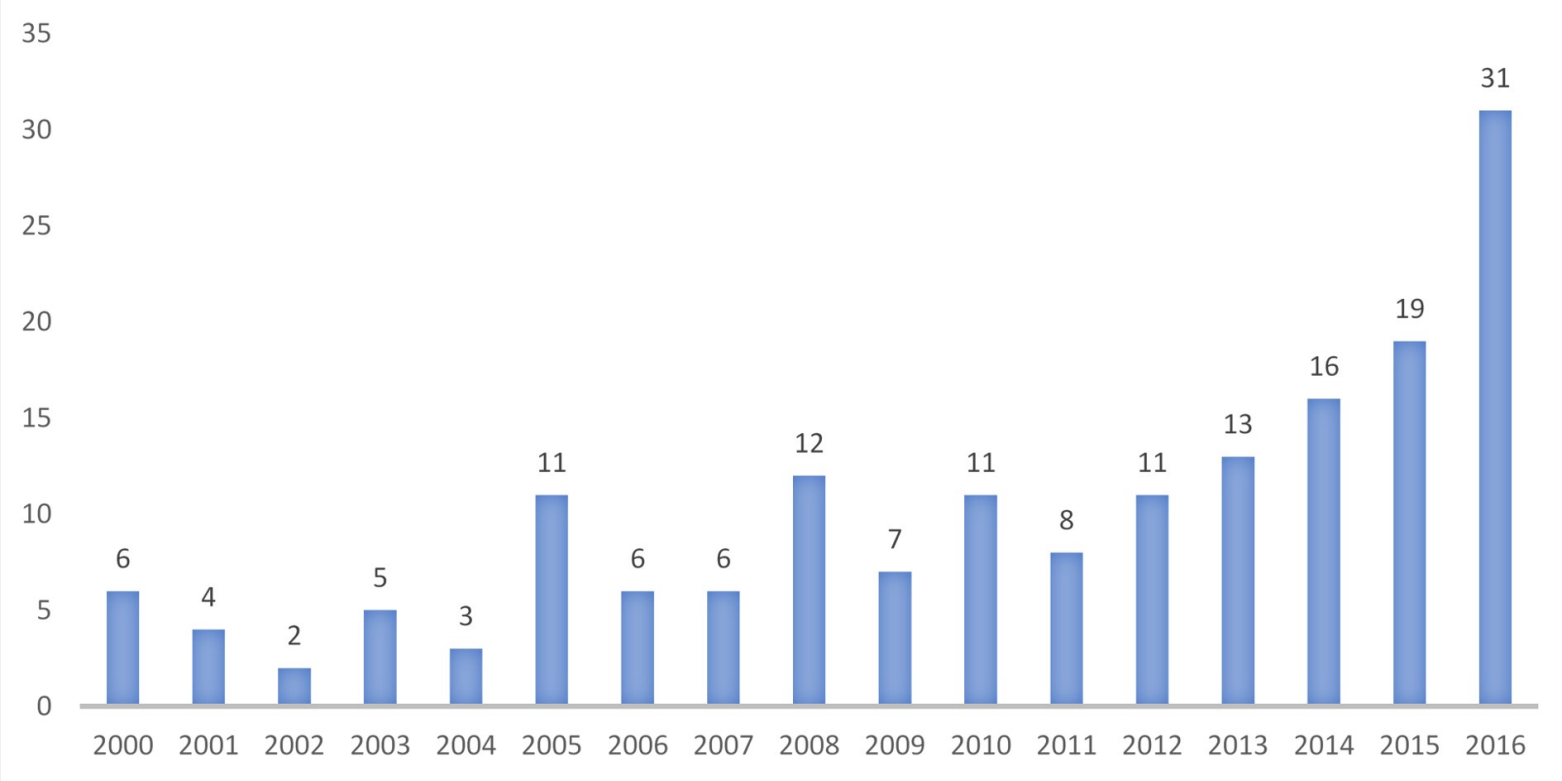
Number of articles per year between 2000 and 2016.

We reviewed 181 studies published between January 1, 2000 and May 31, 2017 that included findings related to AOD use among SMW. As shown in Fig 2, which excludes articles published in 2017 because our review covered only part of that year, research on this topic has grown substantially, particularly since 2012.

**Fig 3:**
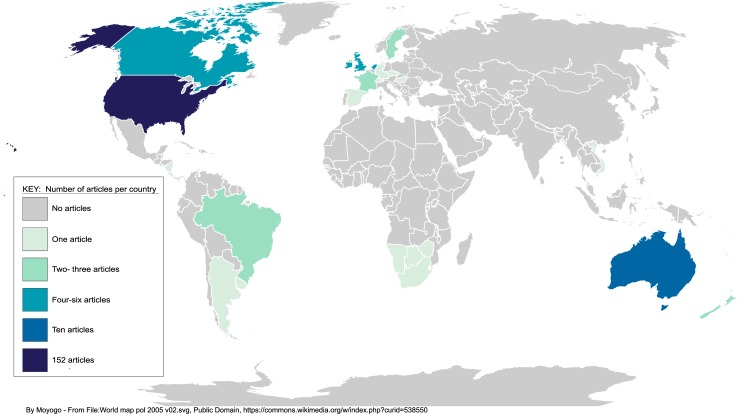
Distribution of articles by country.

There are major gaps in knowledge about SMW’s use of AOD and related problems in most parts of the world. As shown in the map in Fig 3 nearly all studies have been conducted in the US. Ten studies were conducted in Australia, five in the UK, and four in Canada. Our review included one or more studies from Argentina, Botswana, Costa Rica, Denmark, France, Israel, Namibia, Netherlands, New Zealand, Puerto Rico, South Africa, Sweden, Taiwan, and Vietnam. A few studies included data from multiple countries [5,34,35].

### Theoretical framework for understanding heightened risk of AOD use and related problems among SMW

Social determinant frameworks describe how the circumstances in which people live and work shape their health outcomes. These circumstances (i.e., social determinants) are believed to underlie many world health inequalities, such as the greater burden of poor health among disadvantaged populations [36]. Social determinant frameworks build on the concept of the “social gradient.” The social gradient in health refers to the fact that inequalities in population health are related to inequalities in social status [37]. That is, individuals with lower social status (e.g., women, sexual minorities, racial/ethnic minorities) experience greater health risks and poorer health outcomes than those with higher status. Closely aligned with the social determinant framework are theories and perspectives on stigma and minority stress. Explanations for sexual-orientation-related health disparities are largely based on theories of stigma [38]. Until recently, studies of stigma-related stressors among sexual minorities have focused primarily on stressors at the individual and interpersonal levels. For example, Meyer’s minority stress model [39] posits that stigma, prejudice, and discrimination contribute to increased risk of AOD use/misuse and mental health problems among sexual minorities via several primary mechanisms: (1) external, objective stressful events (e.g., discrimination, harassment, and violence); (2) expectations of such events and the vigilance that this requires; (3) the internalization of negative societal attitudes (internalized stigma/homophobia); and (4) rejection sensitivity or stigma consciousness, which often results in concealment of one’s sexual minority status.

Unlike individual and interpersonal forms of stigma, structural stigma has been conceptualized as “societal-level conditions, cultural norms, and institutional policies that constrain the opportunities, resources, and well-being of the stigmatized” [38]. Hatzenbuehler and colleagues [40] found evidence of structural stigma in a study showing that LGBT people living in US counties whose residents strongly opposed same-sex marriage had worse health than those living in counties that were more supportive of same-sex marriage. We conceptualize sexual minority stress as including both individual and institutional forms of prejudice and discrimination that likely contribute to AOD-related health disparities among SMW.

Results of this review are organized to generally reflect these theoretical frameworks. We summarize studies related to prevalence of AOD use among SMW and elaborate on differences across other dimensions of social status (race/ethnicity and age). Although we do not summarize research on AOD use among sexual minority men (SMM; e.g., gay or bisexual men, men who have sex with men or who identify as something other than heterosexual), we mention differences between SMW and SMM in prevalence or correlates of alcohol and drug use when those differences were among the primary findings of a specific study. Consistent with health disparities and stigma frameworks, we also review literature related to risk and protective factors at the individual, interpersonal, and community levels, as well as intersections between AOD use and other health issues.

## Results

### Prevalence of AOD use and related problems

#### Alcohol

Research has consistently found higher rates of alcohol consumption and hazardous drinking (defined by the World Health Organization) [41] as a pattern of alcohol use that increases risk of harmful consequences) among SMW than among heterosexual women. For example, in large US population-based studies such as the National Alcohol Survey (NAS) and the National Epidemiologic Survey on Alcohol and Related Conditions (NESARC) researchers [14,21] found three to seven times higher odds of past-year alcohol dependence among lesbian and bisexual women than among heterosexual women, controlling for age and other relevant demographic variables. Interestingly, sexual-orientation-related differences tend to be more pronounced and consistent among women than men [5,21,42–44]. Sexual minority women are less likely than exclusively heterosexual women (women who identify as heterosexual and who have only had sexual relationships with men) to abstain from alcohol use and more likely to report indicators of hazardous drinking including heavy episodic drinking, drinking to intoxication, and drinking-related problems [14,45]. Studies using behavioral measures of sexual orientation (sex or gender of sexual partners) have also found significantly higher risk of alcohol dependence among SMW than among heterosexual women [46–48]. In addition, SMW appear to be more likely than heterosexual women to report lifetime or past-year treatment, or other help-seeking, for alcohol-related problems [14,45,46].

Studies with smaller, nonprobability samples have found greater alcohol misuse among SMW veterans in the US [49] and higher likelihood of positive screens for alcoholism among SMW participants recruited from women’s groups [50], compared to their heterosexual counterparts.

Research from other countries generally shows the same pattern of alcohol outcomes as studies conducted in the US. Researchers [17] using data from the Australia National Drug Strategy Household Survey found higher rates of high-risk drinking (based on AUDIT-C scores), daily drinking, and ever having attended treatment among lesbian/bisexual women than heterosexual women. Research using one wave of data from the Australian Longitudinal Study on Women's Health (ALSWH) [16,51] found that SMW were more likely than exclusively heterosexual women to be high-risk drinkers. In one of the only studies to combine survey data from multiple (n = 14) countries, Bloomfield and colleagues [34] found that in North America, but not other countries, rates of high-volume drinking (heavy episodic drinking on at least one occasion in the previous month) and risky single occasion drinking were higher among women with same-sex partners than among those with only male partners. King and colleagues [5] conducted a meta-analysis of findings from multiple studies of mental health, substance use, self-harm, and suicide among sexual minorities published between 1997 and 2004. The studies were conducted in seven countries in North America, Europe, and Australasia, with most from the US (17/25, 68%). Relative risk of past-year AOD dependence was 1.5 times higher among LGB people than among heterosexuals. Risk of past-year AOD dependence was particularly high among lesbian and bisexual women (4 times higher than heterosexual women for alcohol dependence and 3.5 times higher for drug dependence).

Although researchers often aggregate data across sexual minority sub-groups (e.g., combining lesbian women and gay men, or lesbian and bisexual women), there is growing consensus that it is important to examine differences across SMW subgroups. For example, in studies that compare SMW and heterosexual women, researchers have found higher rates of heavy drinking or alcohol-related problems among bisexual women, women who self-identify as “mostly heterosexual,” and women who identify as heterosexual but report same-sex partners [14,16,45,52–56]. Analyses of data from the third wave of the 20-year longitudinal Chicago Health and Life Experiences of Women (CHLEW) study [57], which included a large non-probability sample of SMW originally recruited in the Chicago Metropolitan area, showed no difference in drinking outcomes between bisexual- and lesbian-identified women. This finding contrasts with those of the great majority of the studies we reviewed that found higher risk among bisexual women regardless of which of the three major dimensions of sexual orientation (identity, attraction or behavior) was assessed.

*Other drugs*. As with alcohol, estimates of drug use and dependence vary based on how sexual orientation is defined—and findings tend to be more consistent and sexual-identity related differences more pronounced among SMW than SMM [21–23,35,47,54,58–60]. Studies using national or other large probability samples have documented higher rates of marijuana and illicit drug use among SMW than heterosexual women, as well as greater risk of drug dependence or any substance use disorder [16,21–23,47,52,53,58,61–64]. For example, population-based studies conducted in Australia and the US showed three to five times greater odds of past-year marijuana use and two to three times greater odds of other illicit drug use among lesbian and bisexual women compared with exclusively heterosexual women [16,21,23]. Studies using non-probability samples have similarly found elevated rates of drug use and drug-related problems among SMW. For example, studies of drug-using populations [65] and patrons in clubs [66] found that lesbian and bisexual women were more likely than heterosexual women to use club drugs and other illicit drugs. Further, research with community samples of SMW has found unmet needs for AOD treatment [67,68].

Rates of drug use also vary substantially across sexual identity groups, with bisexual women typically showing the highest risk [16,23,35,63]. For example, Paquette and colleagues’ study [63] of sexually active adults in Britain found higher odds of drug use among bisexual (but not lesbian) women than heterosexual women. In contrast, using U.S. nationally representative data McCabe and colleagues [21] found that, compared with heterosexual women, rates of past-year marijuana and illicit drug dependence were higher among lesbian women but not bisexual women. A multi-country study found higher odds of lifetime use of 11 different drugs among bisexual women and men relative to heterosexual women and men [35].

Studies using expanded measures of sexual identity have generally found higher rates of marijuana or other drug use among women who identify as mostly heterosexual [53,69], or who identify as heterosexual but report same-sex partners [23,54,56], than among exclusively heterosexual women.

### Racial/Ethnic differences in AOD use

Although findings about the influence of race/ethnicity on substance use among SMW were somewhat mixed, studies that compared SMW of color and White SMW typically found either no differences, or higher risk of AOD use/misuse among SMW of color. In wave 1 of the longitudinal CHLEW study, Hughes and colleagues [70] found that Black SMW reported fewer lifetime problem drinking consequences and dependence symptoms than White or Latina SMW but were two to three times as likely to report 12-month heavy drinking and almost twice as likely as White SMW to report 12-month problem drinking consequences. Black SMW were half as likely as White SMW to have ever wondered if they were developing an alcohol problem. Using the same dataset, Parks and Hughes [71] found that Black SMW were significantly more likely than White SMW to be past 30-day heavy drinkers. No differences were found in either study in comparisons of Latina and White women. In analyses of data from wave 3 of the CHLEW study, which included more than 700 SMW, women of color reported significantly more past year symptoms of potential alcohol dependence than White women but were less likely to have ever been treated for problems related to AOD use [68].

In a study using data from a large community-based health center in Boston, Massachusetts, Mereish and Bradford [72] found that White SMW had significantly higher odds of reporting lifetime substance use problems than their heterosexual counterparts. SMW of color (Latina and Black participants were combined) had more than four times the odds of reporting lifetime substance use problems as their heterosexual counterparts. Balsam and colleagues [73] examined racial/ethnic differences in one wave of an online longitudinal study of 18- to 25-year-old SMW. No racial/ethnic differences were found in heavy episodic drinking (past-year consumption of 4+ drinks in a day), alcohol problem consequences, or current marijuana use. In another online study of young adult women (ages 18 to 35), Lewis and colleagues [74] found that Black lesbian women reported significantly lower levels of hazardous drinking (based on AUDIT scores) than White lesbian women. MacCarthy and colleagues [75] examined substance use behaviors among Black women presenting for STI or HIV testing. Compared to their heterosexual counterparts, Black SMW reported significantly higher rates of every substance use outcome, including heavy episodic drinking, lifetime marijuana use, crack or cocaine use, and other drug use. Similarly, in the Multi-Site Women’s Health Study conducted in several US cities, Hughes and colleagues [76] found that Black lesbian women were significantly more likely than Black heterosexual women to report current drinking and more than one alcohol-related problem. They also reported higher rates of use of all categories of drugs, although only use of hallucinogens and opiates was significantly higher. Further, twice as many Black lesbian (20%) as Black heterosexual (10%) women reported ever having sought help for a drug or alcohol problem.

We found few studies that examined AOD use among Latina SMW. Only one study included a heterosexual comparison group but did not disaggregate findings from Latina and Asian participants. Cochran and colleagues [77] found that Latinx and Asian SMW were more likely than their heterosexual counterparts to report a recent drug use disorder. Two studies focusing on discrimination had mixed findings. In a community-based sample of Latina SMW, Matthews and colleagues [78] found that more than 70% had ever used marijuana, 19% were heavy drinkers, 36% were current smokers, and more than 30% had ever used cocaine, crack, crystal meth, or heroin. Acculturation and discrimination (based on race/ethnicity, sexual identity, gender, or gender identity) were each independently and significantly associated with substance use; the association between acculturation and substance use was partially mediated by discrimination. In unadjusted analyses of data from a national convenience sample of Latina immigrant SMW, Cerezo [79] found that discrimination based on ethnic minority status and on sexual minority status were significantly associated with substance use. However, these associations were attenuated and no longer significant when adjusting for age, income, education, and acculturation. In the wave 1 CHLEW study described above, Latina women did not differ significantly from White women on any of the alcohol outcomes [76]. However, in a later wave (wave 3) of the study that included a larger and more diverse sample of SMW, Latina (11%), and African American (8%) women had significantly higher rates of past-year alcohol dependence symptoms than white women (4%) [68].

Even fewer studies have examined AOD among Native American or Asian SMW. In a study of lesbian, gay, and bisexual (two-spirit) Native Americans, Yuan and colleagues [80] found that 72% of men and 62% of women engaged in hazardous drinking; 51% of men and 49% of women met criteria for past-year alcohol dependence. In the only study of AOD use among Asian SMW (other than Cochran and Mays’ study of Latina and Asian SMW described above) Dibble and colleagues analyzed data from a sample of midlife Asian, Pacific Islander, and Native Hawaiian lesbian women [81]. Rates of daily drinking were relatively low (7%); 30% reported drinking once a month or a couple times a year, and 11% reported past alcohol-use-related problems. The study did not include a heterosexual comparison group.

Although findings regarding the influence of race/ethnicity on AOD use among SMW were mixed, there is some evidence of elevated risk among SMW of color relative to their heterosexual counterparts.

#### Age-related differences in AOD use

Several articles in this review compared rates of AOD use in younger and older SMW. The most common finding was that rates of AOD use, and hazardous drinking (e.g., heavier drinking, heavy episodic drinking, symptoms of potential problem drinking or alcohol dependence), were higher among younger than older SMW [70,82,83]. Similarly, several studies found that differences in AOD use between SMW and heterosexual women were greater among younger than older women [59,82,84]. Further, in contrast to findings from women in the general population that document declines in drinking among older women, Hughes and colleagues [70] found relatively little age-related variation in lifetime or 30-day abstention rates across four age groups of lesbian women in the CHLEW study. Using data from the same study, Parks and Hughes [71] found that age differences in heavy drinking varied across racial/ethnic groups: among African-American lesbian women, rates of past-12-month heavy drinking were highest in the oldest cohort, whereas among White and Latina lesbian women 12-month heavy drinking was highest in the youngest age cohort. In an online survey of more than 1000 lesbian women, ages 19 to 50 or older, in the Southern US, Austin and Irwin [82] found that problematic alcohol use (defined using CAGE scores) was highest in the youngest age group and decreased with age.

A substantial number of studies have focused on emerging and early adult SMW (age ≤ 30) [16,22,59,61,73,85,86]. Studies that included comparison groups consistently found that young SMW had higher rates of most AOD-related outcomes than young heterosexual women [16,22,59,61]. Other findings included possible resilience of young SMW of color; associations between AOD use and sexual risk behaviors; reciprocal associations between SMW-specific perceived drinking norms and actual alcohol consumption; and greater disparities in AOD use and its mental health correlates among younger than older SMW [22,61,73,85,86].

A smaller number of studies examined AOD use/abuse patterns in older (age 50+ or 60+) SMW [83,87–89]. Only one of these studies included a heterosexual comparison group: Fredriksen-Goldsen and colleagues [88] found that older SMW were more likely than older heterosexual women to smoke and to drink excessively (defined as four or more drinks/occasion in the previous month). Masini and Barrett [89] found that alcohol use (1+ drinks in past 30 days) was considerably higher in SMW (73% of lesbian and bisexual women combined) than national norms for same-aged women (49%). Other findings were that older lesbian women had higher rates of excessive drinking than older bisexual women; non-Hispanic White SMW had higher rates of high-risk drinking than did SMW of color; and scores on screening instruments for alcohol abuse and drug abuse correlated negatively with time spent with other sexual minority people [83,87,88].

### Risk and protective factors

#### Minority stress

Even though minority stress is considered a major driver of sexual-orientation-related health disparities [39], relatively few studies directly tested the links between minority stressors and substance use among SMW. D’Augelli and Grossman in 2001 [87] were among the earliest researchers to examine associations between stress associated with sexual minority status and alcohol use and mental health. The study was also unique in that it focused on older (ages 60 to 91 years) lesbian, gay, and bisexual people. Alcohol or drug use did not appear to be significant predictors of mental health in this older sample.

Many studies that examined relationships between AOD use and minority stress focused on sexual-orientation-based discrimination or internalized homophobia. For example, in analyses of NESARC data, Lee and colleagues [90] found that more than half of SMM and nearly half of SMW reported lifetime experience of sexual orientation-based discrimination. The relationships between discrimination and substance use outcomes were mixed and significant only among men. Using the same dataset but expanding discrimination to also include experiences based on sex and race/ethnicity, McCabe and colleagues [91] found that substance use disorders (SUDs) were more prevalent among LGB respondents who reported any of the three forms of discrimination compared with those who reported no discrimination. Prevalence was highest among LGB respondents who reported all three types of discrimination. In analyses of data from the Swedish Twin Study, Frisell and colleagues [92] found that women in same-sex relationships had three times greater odds of alcohol dependence than their twin sisters who were in opposite-sex relationships. Discrimination explained some, but not all, of the variance in alcohol dependence. In a rare quasi-experimental study, Everett, Hatzenbuehler, and Hughes [93] examined the impact of new civil union legislation in Illinois (a mid-Western state in the US) on minority stress, depression, and hazardous drinking in wave 3 of the CHLEW. Legislation was associated with reductions in stigma consciousness, perceived discrimination, depressive symptoms, and adverse drinking consequences in the full sample. SMW of color and those with high school education or less showed greater reductions in stigma consciousness, suggesting that policies supportive of sexual minorities may disproportionately benefit the most vulnerable SMW. In 2006 Amadio [94] found partial support for a positive relationship between internalized heterosexism and alcohol use among SMW, but not SMM. In an online study of 18- to 25-year-old same-sex attracted women and men in Australia, Lea and colleagues [95] found overall low levels of internalized homophobia and perceived stigma. SMW were less likely than SMM to report past-month club drug use. There were no significant sex differences in psychological distress or club drug dependence and no significant sexual identity differences in any of the mental health or substance use outcomes. The associations between minority stress and substance use were inconsistent. Austin and Irwin [82] found general risk factors (depression and stress) were more strongly associated with alcohol-problem symptoms than were discrimination, lesbian/gay related stigma, or internalized homophobia.

Lehavot and Simoni [96] recruited 1,381 lesbian and bisexual women for an online survey that assessed gender expression, minority stressors (victimization, internalized homophobia, and concealment), social–psychological resources (social support, spirituality), and health-related outcomes (substance use and mental health). Higher levels of masculine/butch gender expression were associated with greater LGB victimization but with less internalized homophobia and concealment. Each of the minority stressors was negatively associated with social–psychological resources, which was negatively associated with mental health and substance use. In addition, LGB victimization was also directly associated with substance use. This model accounted for 56% of the variance in mental health problems and 14% of the variance in substance use.

Other studies [97–101] examined the relationships between substance use and minority stressors of various forms such as heterosexist events, and guilt-proneness and shame-proneness. Another study [102] examined how early drinking onset, minority stress, and drinking self-schemas influence substance use. These studies showed mixed support for the association between minority stress and substance use. Finally, following the Pulse nightclub shooting in Florida, Boyle and colleagues [103] found that the odds of using AODs to cope with the event were higher among SMs who perceived that their peers used alcohol or drugs to cope with the event than those who did not hold this perception.

#### Sexual orientation disclosure

The small number of studies that examined the association between sexual orientation disclosure and AOD use reported mixed findings. Feinstein, Dyer, and London [104] analyzed data from SMW recruited online and found that higher levels of sexual orientation disclosure were associated with greater numbers of AOD-use related consequences among bisexual women, but not among lesbian- or queer-identified women. In a sample of lesbian women in New Zealand, Saphira, and Glover [105] found no association between level of disclosure and alcohol use; however, women who reported marijuana use reported higher levels of disclosure. Rothman and colleagues [106] examined disclosure and others’ reactions to disclosure in a sample of LGB participants in the 2002 Massachusetts Behavioral Risk Factor Surveillance Survey. Most (73%) LGB participants had disclosed their sexual identity to their parents (or stepparents). Two-thirds of these participants reported receiving what they perceived as adequate social and emotional support. Among SMW, non-disclosure was associated with 12 times the odds of past-month illicit drug use and 1.5 times the odds of binge drinking (5+ drinks/occasion in the past month). Lesbian and bisexual women who disclosed their sexual identity and whose parents were not supportive were more than 11 times as likely as those with supportive parents to report lifetime illicit drug use. Similarly, in a study [107] of family reactions to SMW’s disclosure conducted in Vietnam, negative reactions (e.g., beating up the participant and/or her partner, calling the police, having the participant locked up) were associated with significantly higher risk of past-month heavy drinking compared to those whose familial reactions were positive or neutral.

#### Violence and victimization

Although a relatively large number of studies focused on links between violence/victimization and AOD use in SMW, only one [109] explicitly examined victimization experiences that occurred because of sexual minority status. Nevertheless, studies in this review indicate that SMW are more likely than exclusively heterosexual women to report nearly every form of childhood and adulthood victimization, and that victimization is generally positively associated with AOD-use outcomes. For example, Drabble and colleagues [108] used data from more than 1100 women in the US National Alcohol Surveys to compare relationships between hazardous drinking and sexual and physical abuse by sexual identity. Rates of lifetime victimization were higher in each of three SMW subgroups (lesbian, 59%, bisexual, 76%, and heterosexually-identified women who reported having same-sex partners, 64%) than in exclusively heterosexual women (42%). Women who reported childhood physical abuse (CPA) were 50% more likely, and women who reported childhood sexual abuse (CSA) were 80% more likely, than those with no childhood victimization to report hazardous drinking. Adult victimization was also strongly predictive of hazardous drinking. In another study using a US national probability sample, Hughes, McCabe and colleagues [15] found that lesbian and bisexual women were significantly more likely than heterosexual women to report any lifetime victimization and to meet criteria for AOD dependence. Among women who reported childhood neglect, those who identified as lesbian had 30 times higher odds of alcohol dependence than heterosexual women.

Balsam, Lehavot, and Beadnell [49] found that lesbian women were significantly more likely than heterosexual women (and gay men) to report CSA and adult rape. SMW who reported CSA and/or adult rape also reported significantly higher levels of alcohol, marijuana, and other drug use than those who reported no victimization. In analyses controlling for CSA, differences in adult rape were no longer statistically significant, leading the authors to speculate that lesbian women’s higher rates of CSA might explain their greater risk of rape in adulthood. In a clinic-based US study, researchers at Fenway Health Center [109] in Boston, Massachusetts, examined substance use problems using a single-item measure (“In your lifetime have you ever felt you had a problem with substance use?”). They found that SMW were more likely than heterosexual women to report childhood abuse, but not intimate partner violence (IPV). The relationship between minority sexual identity and substance use was strongly influenced by childhood abuse and IPV in men, but not in women. Using the same clinical sample, Merish, O’Cleirigh, and Bradford [110] found that SMW who reported LGBT-based victimization had significantly higher odds of a lifetime substance use problem than those who did not report victimization. As in many or most of the studies reviewed, elevated risk of substance use in this study was more apparent in women than in men. In another analysis of Fenway client data [111], CSA, IPV, substance use, and history of suicide attempt were strongly interrelated among SMW.

Bimbi and colleagues [112] found that SMW who reported physical IPV were more likely than women without histories of IPV to report recent cocaine use; women who reported non-physical violence were more likely to report recent alcohol and marijuana use. A large study [113] of LGB people recruited using street intercept survey methods found no difference in rates of mutual IPV (both partners engage in IPV) among lesbian/bisexual women. Other studies [114–116] have found that childhood abuse, IPV, and violent victimization are more prevalent among drug-using SMW than their heterosexual counterparts. Further, Mattocks and colleagues [117] found that SMW veterans were significantly more likely than heterosexual women veterans to report both childhood sexual trauma and military sexual trauma—and to be hazardous drinkers.

Several studies of links between victimization and alcohol use in this review are from the longitudinal CHLEW study. In a CHLEW pilot study, Hughes, Johnson, and Wilsnack [118] found that lesbian women were more likely than heterosexual women to report CSA, but rates of adult sexual assault (ASA) were similar in the two groups. In analyses of wave 1 of the CHLEW, Hughes and colleagues [119] found that CSA directly predicted lifetime alcohol abuse, and CPA directly predicted lifetime psychological distress. CSA also indirectly increased the risk of lifetime alcohol abuse through its negative effect on age at first heterosexual intercourse. CPA had only indirect effects on lifetime alcohol abuse through its strong relationship to lifetime psychological distress. In analyses using a combined data set (2001 National Study of Health and Life Experiences of Women [NSHLEW] and 2001 [wave 1] CHLEW) Hughes and colleagues [120] found that exclusively lesbian, mostly lesbian, and bisexual women were more likely than exclusively heterosexual or mostly heterosexual women to report CSA; women who reported both childhood and adult sexual victimization reported significantly higher levels of hazardous drinking than those without such histories. In the same combined dataset, exclusively and mostly heterosexual women reported fewer types of victimization experiences than bisexual, mostly lesbian, and lesbian women [121]. The latter three groups also reported higher rates of re-victimization than exclusively heterosexual women. The odds of hazardous drinking increased incrementally by 20% for each type of victimization reported.

Lewis and colleagues [122–124] are among the few researchers who have examined associations between hazardous drinking and IPV among SMW. Among this group’s findings are that (1) hazardous drinking among lesbian women was significantly associated with physical IPV; (2) greater emotional distress was associated with increased drinking to cope, which was in turn associated with a higher number of maximum drinks, which predicted increased likelihood of bi-directional partner violence; and (3) lesbian women who reported discrepant alcohol use between themselves and their partners also reported poorer relationship quality after controlling for psychological and physical aggression. In a study of SMW in southern Africa, Sandfort and colleagues [125] found that forced sex by women was more likely to involve intimate partners than forced sex by men. Forced sex by men was significantly associated with drug-use-related problems whereas forced sex by women was associated with drinking-related problems.

Using population-based data from the psychiatric healthcare and prescription drug records of nearly 31,000 individuals in the Stockholm, Sweden, Public Health Cohort, Bränström [126] showed that bisexual women were nearly twice as likely as heterosexual women to receive AOD treatment. More frequent experiences of victimization/threat of violence and lack of social support partially explained these disparities. Hequembourg and colleagues [127] also found that bisexual women showed greater risk than lesbian women of sexual or physical assault and negative alcohol- or drug-use outcomes. Talley and colleagues [69] found that mostly heterosexual women were more likely than exclusively heterosexual women to report CPA, lifetime tobacco, and marijuana use, past-year symptoms of alcohol-use disorder, and recent marijuana use; they were more likely than bisexual women to have ever tried marijuana. An online study [73] of 18- to 25-year-old SMW found that African American SMW had higher odds, and Asian American SMW had lower odds, of reporting CSA than White SMW. Asian American SMW also had lower odds of adult rape and lower peak drinking. A study [80] of two-spirit persons found that about half of the sample met criteria for alcohol dependence; among past-year women drinkers the most common type of childhood maltreatment was emotional abuse (72%).

#### Intimate relationships

There has been little research on the associations between hazardous drinking and relationship factors among SMW. Generally, findings suggest that relationships are important in understanding risk and protective factors for hazardous alcohol use. Hazardous drinking among SMW, particularly when in relationship with a partner who does not drink or drinks moderately, appears to predict poor relationship outcomes [124]. Using data from the same sample, Mason and colleagues [122] found that hazardous drinking was significantly associated with IPV perpetration.

Partner sex/gender may also be associated with substance use outcomes. Molina and colleagues [128] found that bisexual women with a single male partner reported significantly more alcohol problem consequences and higher odds of binge drinking than those with a single female partner. Further, compared to women with a single female or male partner, those with multiple partners reported a greater number of alcohol consequences, but similar levels of binge drinking. Results of mediation analyses indicated that internalized bi-negativity explained the higher levels of alcohol. Reczek, Liu, and Spiker [129] found similar levels of moderate and heavy drinking among women in cohabiting relationships, irrespective of sex of partner.

#### Religion/Religiosity

There was been very little quantitative research examining the association between religion and substance use among SMW. In one exception, Drabble and colleagues [130] examined data from a large sample of US women who participated in one of three population-based National Alcohol Surveys (2000, 2005, 2010). Religiosity (the importance of religion in one’s life) was significantly greater among exclusively heterosexual women than among lesbian or bisexual women or heterosexual women who reported same-sex sexual experience. Lesbian women reported the lowest rates of affiliation with religions/denominations that discourage alcohol use. High religiosity was associated with lifetime alcohol abstention and was found to be protective against hazardous drinking and drug use among both SMW and heterosexual women. Reporting religious norms unfavorable to drinking was protective against hazardous drinking among exclusively heterosexual women but not SMW.

#### Drinking contexts and drinking norms

Early studies [131] used “gay-friendly” bars to recruit large enough samples to examine drinking patterns among sexual minorities, resulting in artificially inflated estimates of heavy drinking and drinking-related problems. Subsequently, research with less biased samples has provided more nuanced insights into social norms and the contexts in which SMW drink. Findings from several studies underscore the complexity of the associations between drinking contexts and hazardous drinking among SMW. For example, a US national population-based study [132] found that SMW (including heterosexually-identified women who reported having one or more same-sex partners in the past five years) patronized bars and attended parties more often than exclusively heterosexual women; bisexual women (but not lesbian women) drank more than exclusively heterosexual women in both bar and party contexts. Two surveys [133,134] using non-probability samples of SMW in different regions of the US examined associations between adult drinking outcomes and retrospective reports of drinking behavior and drinking contexts during early sexual identity development. Findings suggested that hazardous drinking among adult SMW was predicted by heavy drinking in sexual-minority specific bars and social settings. A mixed-methods of study [135] of motivations for bar patronage among sexual minorities and heterosexual individuals found that social motives were among the most common reasons for going to bars. Social motives were not associated with problematic drinking; however, frequenting bars in an attempt to change mood was positively associated with drinking-related consequences and alcohol dependence symptoms. SMW perceive greater alcohol availability in LGB social contexts than non LGB contexts, perceive heavier drinking as normative, and overestimate the quantity of alcohol consumed by peers [133,134,136,137]. More frequent bar attendance appears to be associated with overestimates of how much other SMW drink [137].

### AOD use and other health concerns

#### Mental health

Although there is a wealth of data on the associations between AOD use and mental health among women in the general population, research on this topic with SMW is more limited and findings are inconsistent. In the ALSWH [16], SMW in Australia reported significantly higher levels of stress, depression, and poor mental health than exclusively heterosexual women. Depression predicted drug use other than marijuana, but not drinking outcomes in the full sample of both sexual minority and exclusively heterosexual women [16]. In one of the few longitudinal studies included in the current review, Johnson and colleagues [138] used data from two waves of the CHLEW study. Anxiety was prospectively associated with hazardous drinking, and hazardous drinking was prospectively associated with depression. These findings suggest that SMW may use alcohol to self-medicate symptoms of anxiety and that hazardous drinking may, in turn, lead to depression. Using 2004–2005 NESARC-II data, Mereish and colleagues [139] found that among women with a lifetime alcohol use disorder, SMW had higher prevalence of any mood disorders, dysthymia, and panic disorder than their heterosexual counterparts. In contrast, a study [140] that recruited sexual minorities from LGBT centers in the Los Angeles, California, area found that depression was not significantly associated with drinking frequency among SMW. This study found a significant interaction effect of internalized homophobia and depression on drinking frequency, suggesting that SMW who have high levels of both internalized homophobia and depression may drink more frequently.

Talley and colleagues [141] used NESARC data to examine the contribution of sexual-identity-related stress to risk of AOD dependence in SMW and SMM. Identity disturbance was more common among sexual minorities than heterosexuals and was strongly predictive of lifetime alcohol dependence, illicit drug dependence, and any substance dependence. Associations were more consistent among women than men and were evident among women across the three major dimensions of minority sexual orientation (sexual identity, sexual behavior, and sexual attraction).

#### Suicidal ideation and behavior

Elevated rates of suicidal ideation and behaviors have been consistently documented in sexual minority samples. In a convenience sample of LGB individuals [142], 91% reported ever having suicidal thoughts; of these, 41% had seriously considered suicide and almost one-fourth had attempted suicide. Among SMW, interpersonal abuse, but not AOD use, predicted both suicidal behavior and suicide attempts. Mereish, O’Cleirigh, and Bradford [110] found that history of victimization was significantly associated with history of substance use problems, suicidal ideation, and suicide attempts in a clinical sample of SMW at a primary health care center. Controlling for victimization, women who reported substance use problems had three times higher likelihood of suicidal ideation and five times higher likelihood of suicide attempts. Substance use partially mediated the association between sexual-minority-based victimization and suicidal ideation and attempts. Using data from a probability sample of adults aged 20–24 and 40–44 years (at baseline) living in and around the Australian Capital Territory, researchers [143] found that sexual abuse by a parent had the strongest association with self-harm, followed by bisexual identity. Current marijuana use and alcohol dependence (but not hazardous drinking) were significantly associated with self-harm; ecstasy and amphetamine use were not. The authors did not test whether the associations between substance use and self-harm might differ by sexual identity. In a nationally representative sample from France [144], participants who identified as LGB had significantly higher rates of suicidal ideation, but not attempts. Substance use and physical and sexual assault were significantly associated with suicidality.

#### Physical health

Although the physical health of sexual minorities has received much less research attention than mental health, we found more than 30 studies of physical health among sexual minorities that included assessments of AOD use. All of the studies included measures of alcohol use or alcohol-related problems but only one-third of the studies also assessed drug use. More than one-half of the studies used probability samples. Many of the US studies used Behavioral Risk Factor Surveillance System (BRFSS) data from one or more states in the US. Several studies analyzed data from longitudinal surveys (ALSWH, the National Longitudinal Study of Adolescent to Adult Health [Add Health], and Growing Up Today Study [GUTS]), but only two conducted time-ordered analyses of longitudinal data. In one of these, Hatzenbuehler and colleagues [145] examined stressful life events in four waves of data from Add Health and cardiometabolic biomarkers at wave 4 when participants were 24- to 32-years old. Alcohol use (binge drinking) was included only as a covariate. The other longitudinal physical health study [146] used seven waves of data from GUTS, which recruited 9- to 14-year-old children of women in the Nurse’s Health Study II, a national prospective cohort study of female registered nurses in the US. This study examined cancer-related risk behaviors over time. Rates of binge drinking (defined as drinking four or more drinks in a few hours, six or more times in the previous year) were not statistically different across sexual identities with one exception: women who identified as mostly heterosexual were more likely than their completely heterosexual counterparts to meet criteria for binge drinking. Only one study tested the association between AOD use and a physical health outcome: Everett and Molborn [61] included AOD use as one of the predictors of hypertension among LGB participants in the US Add Health study. In the other studies, AOD use was assessed as one of a variety of outcomes (e.g., smoking, diet, physical activity, obesity), a behavioral health risk factor generally, or a risk factor in relation to a specific health condition. For example, six studies focused on risk factors for cardiovascular disease (CVD) and 10 focused on risk factors for cancer; two studies focused on risk factors for both CVD and cancer. Although these studies of physical health among SMW found few relationships between alcohol use and physical health outcomes, many of them used probability samples and produced prevalence estimates of various AOD use outcomes among SMW which add to overall evidence of sexual-orientation-related AOD use disparities. For example, in the national or multi-state studies [147–150] that disaggregated lesbian and bisexual respondents analyses found greater odds of heavy drinking and binge drinking among both lesbian and bisexual women than among heterosexual women. Some outcomes varied by how subgroups were defined. For example, a study [150] that defined SMW as lesbian, bisexual, or something else/don’t know found nearly two times the odds of heavy drinking (defined as > 1 drink/day) among the “something else/don’t know” women compared to heterosexual women.

#### AOD use and sexual risk

The associations between sexual risk behaviors and AOD use appear to differ by sexual identity and by race/ethnicity. Using two waves of data from the CHLEW, Matthews, Cho, and colleagues [151] found that SMW who reported high levels of sex-related drinking expectancies (e.g., believing that drinking would improve their sexual experiences) at baseline were more likely to be defined as hazardous drinkers in wave 2. Further, hazardous drinking at baseline was associated with increased sexual risk behaviors at wave 2. Bisexual women and Black SMW reported more sexual risk behaviors at wave 2 than lesbian and White women, respectively. In a study of women presenting for STI and HIV screening at a community clinic [75], Black SMW were more than two times as likely as Black heterosexual women to have used AOD—and to have a partner who used AOD—the last time they had sex. In a clinic sample of women recruited at a sexual and gender minority health center, Estrich, Gratzer, and Hotton [152] found that bisexual women were significantly more likely than either lesbian or heterosexual women to report that they and their partner used drugs the last time they had sex, and more likely than lesbian women to report that they and their partner used alcohol the last time they had sex.

In a study of women in Puerto Rico, Soto-Salgado and colleagues [153] found that those who reported ever having a same-sex partner (n = 39; 7%) were more likely than women with only male partners to report lifetime use of illicit drugs and current smoking; no significant differences in rates of alcohol consumption were found. Among women in the 2002 National Survey of Family Growth, Bauer, Jairam, and Baidoobonso [54] found that heterosexually-identified women who reported having a same-sex partner were significantly more likely than lesbian women and exclusively heterosexual women to be daily smokers, weekly drinkers, binge drinkers and to have used marijuana and cocaine in the past year. This group did not differ significantly from bisexual women on tobacco use or from lesbian or bisexual women on weekly drinking.

## Discussion

As shown in Fig 1 in the results section, there was a substantial and linear increase between 2000 and 2017 in the number of research reports that focused on AOD use among SMW. In addition to an increase in the quantity of research, there is evidence of methodological and theoretical improvements in this research. For example, many more studies in the past 10 years have included probability samples and multiple measures of sexual orientation. There has also been a modest increase in longitudinal research designs, in studies that address mediators and moderators, and in the explicit use of theoretical frameworks to guide research aims and hypotheses. Nevertheless, many gaps remain and there is much room for improvement in the quantity and quality of research on AOD use among SMW.

Although the number of studies on AOD use among sexual minorities is growing, inclusion of SMW remains limited because much of this research has focused on HIV risk among men who have sex with men. One reason for this is that funding for sexual minority health research disproportionately addresses sexually transmitted diseases, particularly HIV/AIDS. Between 1989 and 2011, apart from studies of HIV/AIDS, only 0.1% of all studies funded by the US National Institutes of Health focused on sexual minority health. Of these, most focused on men; only 13.5% addressed SMW’s health [33].

In most studies that we reviewed SMW reported higher rates than heterosexual women of nearly every alcohol-related outcome that was assessed. Further, except in a handful of studies, bisexual women showed the highest risk of all sexual orientation subgroups. Although less often assessed, women who identified as mostly or mainly heterosexual—or who identified as heterosexual but reported having had same-sex sexual partners—showed higher risk than exclusively heterosexual women, and in a few studies showed higher risk than lesbian women. In addition, in most studies that included both SMW and SMM, alcohol outcomes were more prominent among SMW. Sex-differences-related patterns of drug use were less clear, largely because relatively little drug research has included SMW. Some of the inconsistency in findings also likely relates to differences in the kind of drugs asked about and how ‘use’ was operationalized in the studies.

Although there is some support for lower or slower age-related maturing out of harmful AOD use among SMW compared with heterosexual women, studies in this review showed that younger SMW were more likely than their older counterparts to report hazardous drinking and drug use. There is also clear evidence that risk decreases with age among SMW. Evidence regarding racial/ethnic differences in AOD use and related outcomes was less consistent—with some studies showing Black SMW at higher risk than White SMW and others showing the opposite. In general, female sex and minority race/ethnicity do not appear to provide the same level of protection against AOD use and related outcomes among SMW as among heterosexual women.

More difficult to summarize are results related to risk and protective factors associated with AOD outcomes among SMW. The bulk of studies showed positive associations between discrimination and AOD outcomes. Findings related to internalized homonegativity/heterosexism were less consistent—with some studies showing strong positive relationships and others failing to find significant associations. One of the most consistently robust findings in the studies reviewed was higher rates of lifetime interpersonal violence and victimization among SMW. Further, violence and victimization were generally significantly associated with AOD outcomes. However, almost no studies assessed whether violence/victimization occurred because of minority status. Therefore, it cannot be determined whether SMW are at greater risk of some forms of violence/victimization because of their sexual minority status.

Consistent with findings in the literature about the influence of intimate relationships on AOD use, we found some evidence that living in a same-sex household makes it more difficult for SMW to conceal their relationship and their sexual identity. Although some have speculated that SMW who are also racial/ethnic minorities may be at greater risk than White SMW of family and community rejection, it appears that religious factors may account for racial/ethnic differences in support of same-sex relationships and that secular influences play less of a role in structuring African Americans' and Latinx’ beliefs about same-sex relationships [154–157].

As in the broader literature on whether disclosure of minority sexual orientation has a positive or negative effect on mental health, findings from the studies we reviewed showed mixed results related to AOD use. The available studies do, however, suggest that the outcome depends on the reactions of those to whom SMW disclose.

### Limitations in research on AOD use among SMW

#### Methodological limitations

Noteworthy methodologic limitations in research on AOD use among SMW include the 1) preponderance of cross-sectional, descriptive research designs; 2) general reliance on non-probability samples that tend to over-represent White, well-educated, and young SMW; 3) wide variations in labeling and defining AOD-related variables; 4) focus on only one dimension of sexual orientation; 5) combining lesbian and bisexual women, or all SMW and SMM, in analyses; and 6) lack of attention to how changes in sexual identity might influence substance use.

Longitudinal designs, although very rare in research on AOD use among SMW, are important for testing mediational mechanisms and necessary in understanding how changes in social determinants impact AOD use. Research on moderators of the links between stress and negative AOD outcomes is needed to better understand why some SMW are more, or less, influenced by exposure to stressors. Such moderators include individual characteristics (e.g., race/ethnicity, coping skills, SES), interpersonal factors (e.g., social support, relationship status), and structural factors (e.g., anti-discriminatory workplace policies). Theory-driven research on moderators is needed to identify SMW who are at particularly high risk for hazardous drinking or drug misuse—as well as to identify modifiable factors that could help reduce the negative impact of stressors. Longitudinal study designs are also important for examining the impact of changes in societal attitudes and of policies and laws (e.g., same-sex marriage) that likely influence the health of sexual minorities.

Although a growing number of AOD-related studies include probability samples, such studies remain the exception. Probability samples (sometimes referred to as random samples) are the gold standard of sampling for survey research because they allow generalization of results to the population from which the sample was drawn. This is necessary in estimating population parameters, such as the prevalence of AOD use and related outcomes among sexual minorities. This is an important area that needs improvement to grow the expanding knowledge base about AOD use among SMW. Currently most studies that include probability samples use existing data from large national surveys. These studies have provided important information about sexual minority health and health disparities, but they are limited in that they rarely include sexual minority-specific risk factors or large enough samples of sexual minority subgroups to permit comparisons based on age, sex, race/ethnicity or other important characteristics. Researchers [158,159] have begun to advocate for oversampling of sexual minority participants to provide large enough subgroups for such comparisons.

Even though it is not possible to know the extent to which findings from non-probability samples accurately characterize the sexual minority population, studies based on nonprobability samples can yield valuable information, especially if findings are replicated in multiple studies [160]. As described above, most probability samples of sexual minorities are limited in size or in the scope of variables included in the study. They are also limited by the unknown extent to which sexual minority participants fail to answer, or answer truthfully, questions about sexual orientation. In addition to providing general descriptive data for sexual minority populations and subgroups, nonprobability samples permit experimental tests of the effectiveness of various behavioral or health interventions; assess relationships among study variables; identify differences among groups; and in general, provide insights into health-related challenges faced by sexual minority populations [1].

Despite growing evidence of both sexual orientation and sex differences in AOD-related outcomes a surprising number of studies combined lesbian and bisexual women or all SMW in analyses that compared SMW and heterosexual women. Decisions to combine lesbian and bisexual women are often made because of small sample sizes or assumptions of similarity but should be discouraged whenever possible [162]. One of the most robust findings in this review was the higher rates of AOD use and related risks among bisexual women compared with other sexual minority and heterosexual women. Similarly, combining SMW and SMM in analyses obscures sex and gender differences that are important in understanding AOD-related risk factors.

### Limitations in measurement of AOD

There was a large amount of variability in how substance use was operationalized in the articles we reviewed. For example, some researchers assessed AOD use over a specific time (e.g., past month, past year, lifetime), others examined risky use (e.g., heavy episodic/binge drinking, intoxication or use-related problems), or self-perception of having or having had AOD-related problems, and a few focused on diagnosable outcomes (e.g., DSM substance use disorders). Developing accepted measurement standards for AOD use and related problems among sexual minority people would permit more accurate cross-study comparisons and more sophisticated methods, such as meta-analytic studies.

The field would benefit from more studies that ask questions beyond lifetime AOD-use related problems, or frequency and quantity of drinking. Greater attention to subjective assessments of intoxication, maximum number of drinks, patterns of high-risk drinking (e.g., drinking in conjunction with driving or sex) and negative consequences (e.g., accidents, heightened risk of violence) provide a more comprehensive assessment of risk beyond traditional consumption measures (e.g., quantity, frequency) [161].

### Limitations in measurement of sexual orientation

Relatively few studies in this review assessed multiple dimensions of sexual orientation—even though it is now well-accepted that sexual orientation includes at least three major dimensions (sexual identity, sexual behavior, and sexual attraction). McCabe and colleagues [21] demonstrated that across all three major dimensions of sexual orientation, non-heterosexual orientation was typically associated with elevated likelihood of substance use and dependence—although the strength of the associations varied. The associations between sexual identity and substance use/dependence in the McCabe study were stronger than the associations between same-sex attraction or behavior and substance use/dependence. In contrast, prevalence rates for substance use/dependence did not vary across the sexual orientation dimensions among heterosexual respondents. McCabe and colleagues [21] also found that regardless of which sexual orientation dimension was examined, effects (sexual minority participants higher than heterosexual participants) were consistently larger among women than men—a common finding in studies of AOD use conducted in various parts of world [13].

Using only one dimension of sexual orientation may underestimate risk among sexual minorities. For example, focusing solely on same-sex relationships or sexual behavior limits the sample to SMW who are in relationships or who have had the opportunity to engage in same-sex behavior. Bauer and Brennan [162] point out another problem with this approach. In most studies, to be counted as bisexual, participants must report at least one male and one female partner within a given timeframe, whereas being counted as lesbian or heterosexual requires only one sexual partner. These researchers used data from the 2002 US National Survey of Family Growth to demonstrate that the observed effects of ‘behavioral bisexuality’ on AOD use and sexual health outcomes may result from differences in the number of sex partners, rather than bisexuality as typically defined. They found that past-year behavioral bisexuality performed poorly as a proxy measure for either bisexual identity or lifetime bisexual behavior. The authors recommend that researchers who use such measures be cautious in their interpretations. And, even though sexual identity (compared with sexual attraction or behavior) appears to be the most robust predictor of AOD use and related problems, many SMW are unable or unwilling to identify as such [21,163]. By assessing multiple dimensions of sexual orientation researchers can examine differences in AOD-related outcomes based on each individual dimension as well as on combinations of dimensions. For example, on some measures of AOD use women who identify as heterosexual but report same-sex partners show similar risks as lesbian and bisexual women [14]. Further, this lack of ‘congruence’ among sexual orientation dimensions appears to be associated with poor health outcomes [55].

Relatively few studies included expanded measures of sexual identity (e.g., response options that include mostly heterosexual). McCabe and colleagues [164] demonstrated the importance of using expanded sexual identity measures in a methodological experiment using data from the Student Life Survey at the University of Michigan in the US. In this study, the researchers randomly included either a 3-category and a 5-category sexual identity question in each survey questionnaire. Approximately one-third of students who identified as bisexual based on the 3-category measure chose ‘‘mostly heterosexual” or ‘‘mostly lesbian/gay” on the 5-category measure. Beyond illustrating the potential misclassification of study participants when estimating the prevalence of various sexual minority subgroups, the researchers found that choice of measure impacted study outcomes. In analyses using the 3-category measure, bisexual participants showed greater risk of AOD use whereas when data were analyzed using the 5-category measure mostly heterosexual participants showed the greatest risk.

Minority sexual orientation among women is often fluid [165–167], a factor ignored in most of the studies we reviewed. Studies have shown that changes in sexual identity are associated with depressive symptoms among adult SMW, and with depression and AOD use among adolescent and young adult sexual minorities. Longitudinal research that permits examination of changes in sexual orientation and how these relate to AOD use across time is needed to better understand this process.

### Theoretical limitations

Until Meyer [39] published a paper describing the minority stress model, few studies on AOD use among SMW mentioned a theoretical model. Although minority stress was described as the guiding theoretical framework or perspective in many of the studies that we reviewed, relatively few tested components of this framework. It is important to recognize that there are multiple pathways to sexual-orientation-related AOD disparities, and that other theoretical perspectives and frameworks can help advance understanding of SMW’s substance use. For example, intersectionality posits that multiple social categories (e.g., race, ethnicity, sex, gender, sexual orientation, socioeconomic status) intersect at the micro level of individual experience to reflect interacting systems of privilege and oppression at the macro, social-structural level (e.g., racism, sexism, heterosexism) [168].

The life course theory or perspective [1] is another approach to understanding SMW’s health. This perspective encompasses ideas and observations from a variety of disciplines including history, sociology, demography, developmental psychology, biology, and economics. It calls attention to the connection between individual lives and the historical and socioeconomic context in which those lives unfold. Similarly, a social ecology conceptual framework incorporates multi-level domains associated with health among SMW [169]. It facilitates the examination of multi-level—structural, social, individual—factors associated with health outcomes that can guide the development of preventive strategies and health promotion interventions.

Other researchers have adopted models that integrate elements of multiple theories. For example, Fredriksen-Goldsen and colleagues [170] use the Health Equity Promotion Model in their research with older LGBT people. This model is based on the premise that all people have the right to achieve their full health potential. It incorporates life course perspectives and considers historical, structural, and environmental contexts, as well as multi-factorial mechanisms (e.g., psychological, social, behavioral, and biological processes) that support or restrict health and well-being.

### Gaps in research on AOD use among SMW

Nearly all research on AOD use among SMW has been conducted in the US. Of the 181 studies reviewed, only 25 (14%) were conducted in countries outside the US. Research is essential for understanding stigma and other social determinants of health in different population groups and subgroups, and in different geographic and cultural contexts. Research is also essential for identifying strategies to reduce such disparities. Science drives policy discussion, formulation, and implementation and evaluations of the needed evidence (e.g., legislation, regulations, and standards of practice that negatively impact SMW) to eliminate health disparities at the local, regional, national, and global levels.

In addition to the lack of research on AOD use among SMW in most parts of the world, we identified many other gaps in our review. For example, there is not yet enough evidence to determine whether SMW of color are at greater or lower risk than White SMW for negative AOD-use outcomes. Even large national surveys rarely include enough racial/ethnic minority SMW to test for racial/ethnic differences. To obtain adequate samples of subgroups based on race/ethnicity, age, or other key demographic characteristics, governments and other funding bodies need to support oversampling of small population groups, such as SMW, that are at high risk for negative health outcomes. Understanding whether and in what circumstances minority race/ethnicity is a risk or protective factor is critically important in designing prevention and intervention strategies that target harmful AOD use.

We also found very few studies that focused on resilience or protective factors. Understanding these factors is essential to the development of prevention and early intervention for AOD use-related problems among SMW. In one of the few studies to examine such factors, Ryan and colleagues [171] found that family acceptance predicted greater self-esteem, social support, and general health status among Hispanic and non-Hispanic White sexual and gender minority young adults. Family acceptance was also protective against depression, substance abuse, and suicidality. Hughes, Szalacha, and McNair [16] found that being married, having children, and having higher levels of education were separately associated with lower odds of risky drinking and illicit drug use among SMW in Australia. More research is needed to understand the impact of institutional policies/programs that promote acceptance of sexual minority people. Research is also needed that examines mediators of the relationship between stressors and health outcomes, such as individual (e.g., coping and resilience) and group (e.g., social support, identification with a sexual minority community) resources and factors that buffer the deleterious effects of stress. There is a need to more systematically examine similarities and differences in factors, such as marriage, that are typically identified as protective against hazardous drinking and drug use among heterosexual women. For example, although affiliation with religions that discourage alcohol use has a protective effect against hazardous drinking among women in general population studies [130,172], this relationship was not evident in a study cited in the current review and religiosity was not protective in a study using data from the CHLEW published after May 2017 [173]. It would be helpful to better understand the effects of religion and religiosity on AOD use among SMW as this may be a key influence on substance use, especially in conservative regions of the world.

Another large gap—perhaps better described as a gaping hole—relates to the lack of intervention studies. None of the studies included in this review tested an intervention, and to our knowledge only one study [174] outside this review has done so. Innovative, resiliency-promoting strategies that target high-risk groups of SMW, such as those who have experienced childhood abuse, are needed to prevent the development of stress-related negative AOD use outcomes. Although we excluded studies that focused on treatment outcomes or barriers to treatment, we did include studies that reported findings related to SMW’s need for treatment [67,68,131]. These and other studies suggest that SMW are not receiving treatment commensurate with their needs [175,176]. In addition, SMW may enter treatment with more severe AOD-related problems than heterosexual women [177]. Information is needed about factors (e.g., discrimination) that are associated with AOD treatment acceptability and engagement among SMW.

### Limitations of this scoping review

In addition to limitations identified in the studies reviewed, we also recognize limitations in our review of this literature. First, the authors are all US-based and therefore our inclusion/exclusion criteria may not reflect the research priorities or cultural contexts of AOD use in other locations. Second, it is inevitable that our search missed some eligible articles. Third, we omitted “gray literature” (e.g., doctoral dissertations, practice guidelines, government reports). Fourth, although we included (and searched deliberately for) studies conducted outside the US, many non-US-based articles were excluded because they used qualitative research designs, non-English language or involved populations defined by HIV/STI risk. Our exclusion of HIV/STI-focused research was intentional. Studies of AOD use among HIV/STI-defined populations constitute a substantial portion of AOD-related research among SMs, and several reviews of this literature have been conducted [178,179]. We also excluded studies about tobacco use. Although cigarette smoking and other tobacco use is an important health behavior among SMW, during the period of our review (2000–2017) there were seven scoping or systematic reviews related to smoking/tobacco use among sexual minorities. For this reason and to narrow the scope of our review we chose to exclude studies of tobacco use unless they also included alcohol or other drugs. Narrowing the focus allowed us to comment on and draw attention to other important segments of the literature. Understanding a broader range of risk and protective factors for AOD use among SMW is a prerequisite for making recommendations for future research and for developing targeted and tailored interventions.

## Summary and recommendations

Our results highlight the field’s interest in minority stress as an important risk factor for AOD use and related outcomes—as well as some advances in the currently available literature. Future directions include continued examination of AOD use in nationally representative samples; use of more sophisticated theoretical frameworks, research designs, and analytic methods; expanded focus on racial/ethnic differences, physical health, older age groups, and resilience/protective factors; improved standardization of sexual orientation, minority stress, and alcohol/drug use measures; more research with sexual minority populations in countries outside the US; and increased efforts to translate what is known about AOD use among SMW into clinical interventions and public health policies.

Compared to research on gay and bisexual men, and men who have sex with men, there has been much less research on substance use among SMW, and almost no studies have used prospective designs. Given the historical changes in the social acceptance of sexual minority people in many parts of the world, longitudinal studies that add new, younger cohorts over time are needed to represent contemporary life experiences, health behaviors, and associated health consequences. Important theoretical developments have occurred over the past decade or two in conceptualizing mechanisms that drive sexual minority health disparities. Some research on mechanisms is underway—although this research has focused less on AOD use than on other health concerns of sexual minorities. Other research opportunities include the use of sibling-comparison designs, inclusion of partners, and family studies.

Methodological innovation is needed in sampling SMW for research and in determining the best questions to use when assessing sexual orientation. For example, although researchers rarely include the intermediate category of ‘mostly heterosexual,’ there is a growing body of literature showing that women who identify in this manner represent the largest proportion of SMW (more than lesbian and bisexual women combined). In addition, women who identify as mostly heterosexual appear to be more like lesbian and bisexual women than exclusively heterosexual women regarding many health-risk behaviors and health concerns, including many related to AOD use. Further, how sexual minority people identify and the labels they use are changing. Increasingly, younger SMW prefer terms such as queer, pansexual or asexual (or multiple identity labels); how these identities relate to AOD-related risk is largely unknown.

Most of the studies reviewed here focused on risks for negative AOD-related outcomes; there has been very little attention to resilience. More research focusing on resilience and protective factors is needed to support the development of early intervention and treatment approaches. Although we must acknowledge that AOD-related disparities based on sexual orientation exist, it is important to remember that most SMW are resilient: despite encountering stigma and discrimination on multiple fronts most do not engage in hazardous drinking or drug use [11]. There are reasons to be optimistic that minority stressors may now play less of a role in AOD use given recent positive changes in social determinants and reductions in structural stigma (e.g., the growing number of countries that have legalized same-sex marriage) [180]. At the same time, continuing shifts in the social and political climate may also serve to amplify minority stressors [181,182].

There is also a great need for research that includes multiple levels of risk and protective factors. Most research on AOD use among SMW has focused on understanding individual contributors to risk (and less frequently protection). In addition, little attention has been paid to structural stigma. In one study [93] using a quasi or natural experimental design investigators compared SMW interviewed before a state policy (structural) change related to legal recognition of same-sex relationships with those interviewed after the change. Women interviewed after the change showed better alcohol use and mental health outcomes than those interviewed before the change.

In summary, marked advances have been made in research on AOD use among SMW, and opportunities are nearly limitless for researchers to make further substantial contributions to advance the science and improve the health of SMW.

## Supporting information

S1 Table PRISMA-ScR checklist. (DOCX)

## Abbreviations and definitions of terms

ADD Health: The National Longitudinal Study of Adolescent to Adult Health (US)
AOD: alcohol or other drugs
AOR: adjusted odds ration
AUD: alcohol use disorder
ALSWH: Australia Longitudinal Study on Women’s Health (Australia)
ASA: adult sexual assault
AUDIT: Alcohol Use Disorders Identification Test
BRFSS: Behavioral Risk Factor Surveillance System (US)
CA: childhood abuse
CHLEW: Chicago Health and Life Experiences of Women study (US)
CINAL: Cumulative Index to Nursing and Allied Health Literature
CPA: childhood physical abuse
CSA: childhood sexual abuse
DAST: Drug Abuse Screening Test
DUD: Drug use disorder
GUTS: Growing Up Today Study (a longitudinal cohort study of adolescent girls and boys living throughout the US)
Exclusively heterosexual: a person who identifies as heterosexual, is attracted to and has only engaged in sexual activity with opposite-sex partners
Heavy drinking: typically defined as 2+ drinks/day or 7 or more drinks per week (for women)
HED: (heavy episodic drinking) definitions vary from 4–6 drinks/day to 4+ drinks/drinking occasion
HIV: Human immunodeficiency virus
LGB: lesbian, gay, bisexual
LGBT: lesbian, gay, bisexual, transgender
MPV: mutual partner violence (sometimes referred to as bi-directional partner violence)
Mostly heterosexual: a person who chooses the response ‘mostly heterosexual’ from options following a question about their sexual identity
NAS: National Alcohol Study (US)
NESARC: National Epidemiologic Survey of Alcohol and Related Conditions (US)
NSHLEW: National Study of Health and Life Experiences of Women (US)
PsycInfo: abstracting and indexing database with more than 3 million records devoted to peer-reviewed literature from the 1800s to the present
PubMed: a free search engine accessing primarily the MEDLINE database of references and abstracts on life sciences and biomedical topics
SM: sexual minority
SMM: sexual minority men
SMW: sexual minority women
STI: sexually transmitted infection
SUD: Substance use disorder
UK: United Kingdom
US: United States
WoS: Web of Science. An online subscription-based scientific citation indexing service originally produced by the Institute for Scientific Information, later maintained by Clarivate Analytics, that provides a comprehensive citation search

## References

[1] Institute of Medicine (IOM) Committee on Lesbian Gay Bisexual and Transgender Health Issues and Research Gaps and Opportunities. The health of lesbian, gay, bisexual, and transgender people: Building a foundation for better understanding Washington (DC): National Academies Press; 2011. 22013611

[2] Caceres BA, Jackman K, Ferrer L, Cato K, Hughes TL. A scoping review of sexual minority women’s health in Latin America and the Caribbean. Int J Nurs Stud. 2019;94:85–97. 10.1016/j.ijnurstu.2019.01.016 30947062 PMC6570531

[3] Daulaire N. The importance of LGBT health on a global scale. LGBT Health. 2014;1(1):8–9. 10.1089/lgbt.2013.0008 26789503

[4] Duvivier RJ, Wiley E. WHO and the health of LGBT individuals. Lancet (London, England). 2015;385(9973):1070–1. 10.1016/S0140-6736(15)60595-5 25797552

[5] King M, Semlyen J, Tai SS, Killaspy H, Osborn D, Popelyuk D, et al A systematic review of mental disorder, suicide, and deliberate self harm in lesbian, gay and bisexual people. BMC Psychiatry. 2008;8(1):70. 18706118 10.1186/1471-244X-8-70 PMC2533652

[6] Muller A, Hughes TL. Making the invisible visible: A systematic review of sexual minority women’s health in Southern Africa. BMC Public Health. 2016;16(1):307. 27066890 10.1186/s12889-016-2980-6 PMC4827176

[7] Regmi PR, Van Teijlingen E. Importance of health and social care research into gender and sexual minority populations in Nepal. Asia Pac J Public Health. 2015;27(8):806–8. 10.1177/1010539515613413 26543163

[8] Yi H, Lee H, Park J, Choi B, Kim SS. Health disparities between lesbian, gay, and bisexual adults and the general population in South Korea: Rainbow Connection Project I. Epidemiol Health. 2017;39:e2017046 10.4178/epih.e2017046 29056030 PMC5790982

[9] Green KE, Feinstein BA. Substance use in lesbian, gay, and bisexual populations: An update on empirical research and implications for treatment. Psychol Addict Behav. 2012;26(2):265 10.1037/a0025424 22061339 PMC3288601

[10] Hughes TL. Alcohol use and alcohol-related problems among lesbians and gay men. Annu Rev Nurs Res. 2005;23:283 16353369

[11] Hughes T. Alcohol use and alcohol-related problems among sexual minority women. Alcoholism Treatment Quarterly. 2011;29(4):403–35. 10.1080/07347324.2011.608336 22470226 PMC3315841

[12] Hughes TL, Eliason M. Substance use and abuse in lesbian, gay, bisexual and transgender populations. J Prim Prev. 2002;22(3):263–98.

[13] Hughes TL, Wilsnack SC, Kantor LW. The influence of gender and sexual orientation on alcohol use and alcohol-related problems: Toward a global perspective. Alcohol Research: Current Reviews. 2016;38(1):121–32. 27159819 10.35946/arcr.v38.1.15 PMC4872607

[14] Drabble L, Midanik LT, Trocki K. Reports of alcohol consumption and alcohol-related problems among homosexual, bisexual and heterosexual respondents: Results from the 2000 National Alcohol Survey. J Stud Alcohol. 2005;66(1):111–21. 10.15288/jsa.2005.66.111 15830911

[15] Hughes T, McCabe SE, Wilsnack SC, West BT, Boyd CJ. Victimization and substance use disorders in a national sample of heterosexual and sexual minority women and men. Addiction. 2010;105(12):2130–40. 10.1111/j.1360-0443.2010.03088.x 20840174 PMC3006226

[16] Hughes T, Szalacha LA, McNair R. Substance abuse and mental health disparities: Comparisons across sexual identity groups in a national sample of young Australian women. Soc Sci Med. 2010;71(4):824–31. 10.1016/j.socscimed.2010.05.009 20579794

[17] Roxburgh A, Lea T, de Wit J, Degenhardt L. Sexual identity and prevalence of alcohol and other drug use among Australians in the general population. Int J Drug Policy. 2016;28:76–82. 10.1016/j.drugpo.2015.11.005 26691433

[18] Drabble L, Trocki K. Alcohol consumption, alcohol-related problems, and other substance use among lesbian and bisexual women. J Lesbian Studies. 2005;9(3):19–30. 10.1300/J155v09n03_03 17548282

[19] Nolen-Hoeksema S. Gender differences in risk factors and consequences for alcohol use and problems. Clin Psychol Rev. 2004;24(8):981–1010. 10.1016/j.cpr.2004.08.003 15533281

[20] Allen NE, Beral V, Casabonne D, Kan SW, Reeves GK, Brown A, et al Moderate alcohol intake and cancer incidence in women. J Natl Cancer Inst. 2009;101(5):296–305. 10.1093/jnci/djn514 19244173

[21] McCabe SE, Hughes TL, Bostwick WB, West BT, Boyd CJ. Sexual orientation, substance use behaviors and substance dependence in the United States. Addiction. 2009;104(8):1333–45. 10.1111/j.1360-0443.2009.02596.x 19438839 PMC2975030

[22] Needham BL. Sexual attraction and trajectories of mental health and substance use during the transition from adolescence to adulthood. J Youth Adolesc. 2012;41(2):179–90. 10.1007/s10964-011-9729-4 22076077

[23] Trocki KF, Drabble LA, Midanik LT. Tobacco, marijuana, and sensation seeking: Comparisons across gay, lesbian, bisexual, and heterosexual groups. Psychol Addict Behav. 2009;23(4):620 10.1037/a0017334 20025368 PMC2801062

[24] Batalla A, Bhattacharyya S, Yuecel M, Fusar-Poli P, Crippa JA, Nogue S, et al Structural and functional imaging studies in chronic cannabis users: A systematic review of adolescent and adult findings. PLoS One. 2013;8(2):e55821 10.1371/journal.pone.0055821 23390554 PMC3563634

[25] Marijuana: How can it affect your health? [Web page]. Atlanta: Centers for Disease Control and Prevention; 2018 [updated February 27, 2018. Available from: https://www.cdc.gov/marijuana/health-effects.html.

[26] Memedovich KA, Dowsett LE, Spackman E, Noseworthy T, Clement F. The adverse health effects and harms related to marijuana use: An overview review. CMAJ open. 2018;6(3):E339 10.9778/cmajo.20180023 30115639 PMC6182105

[27] National Academies of Sciences E, Medicine. The health effects of cannabis and cannabinoids: The current state of evidence and recommendations for research: National Academies Press; 2017. 28182367

[28] Tricco AC, Lillie E, Zarin W, O'Brien KK, Colquhoun H, Levac D, et al PRISMA extension for scoping reviews (PRISMA-scr): Checklist and explanation. Ann Intern Med. 2018. 10.7326/M18-0850 30178033

[29] Arksey H, O'Malley L. Scoping studies: Towards a methodological framework. Int J Soc Res. 2005;8(1):19–32.

[30] Levac D, Colquhoun H, O'Brien KK. Scoping studies: Advancing the methodology. Implementation science. 2010;5(1):69. 20854677 10.1186/1748-5908-5-69 PMC2954944

[31] Kidd JD, Jackman KB, Wolff M, Veldhuis CB, Hughes TL. Risk and protective factors for substance use among sexual and gender minority youth: A scoping review. Curr Addict Rep. 2018;5(2):158–73. 10.1007/s40429-018-0196-9 30393591 PMC6214200

[32] Boehmer U. Twenty years of public health research: Inclusion of lesbian, gay, bisexual, and transgender populations. Am J Public Health. 2002;92(7):1125–30. 10.2105/ajph.92.7.1125 12084696 PMC1447202

[33] Coulter RWS, Kenst KS, Bowen DJ. Research funded by the national institutes of health on the health of lesbian, gay, bisexual, and transgender populations. Am J Public Health. 2014;104(2):e105–e12. 10.2105/AJPH.2013.301501 24328665 PMC3935708

[34] Bloomfield K, Wicki M, Wilsnack S, Hughes T, Gmel G. International differences in alcohol use according to sexual orientation. Subst Abus. 2011;32(4):210–9. 10.1080/08897077.2011.598404 22014251 PMC3319346

[35] Demant D, Hides L, Kavanagh DJ, White KM, Winstock AR, Ferris J. Differences in substance use between sexual orientations in a multi-country sample: Findings from the Global Drug Survey 2015. Journal of Public Health. 2016;39(3):532–41. 10.1093/pubmed/fdw069 27519959

[36] Commission on the Social Determinants of H. Closing the gap in a generation: Health equity through action on the social determinants of health. Geneva: World Health Organization; 2008. Report No.: N/A.

[37] Kosteniuk JG, Dickinson HD. Tracing the social gradient in the health of Canadians: Primary and secondary determinants. Soc Sci Med. 2003;57(2):263–76. 10.1016/s0277-9536(02)00345-3 12765707

[38] Hatzenbuehler ML, Link BG. Introduction to the special issue on structural stigma and health. Soc Sci Med. 2014. 10.1016/j.socscimed.2013.12.017 24445152

[39] Meyer IH. Prejudice, social stress, and mental health in lesbian, gay, and bisexual populations: Conceptual issues and research evidence. Psychol Bull. 2003;129(5):674–97. 10.1037/0033-2909.129.5.674 12956539 PMC2072932

[40] Hatzenbuehler ML, Flores AR, Gates GJ. Social attitudes regarding same‐sex marriage and LGBT health disparities: Results from a national probability sample. J Soc Issues. 2017;73(3):508–28.

[41] Lexicon of alcohol and drug terms published by the World Health Organization [Internet]. 1994 [cited 2019 August 1]. Available from: https://www.who.int/substance_abuse/terminology/who_lexicon/en/.

[42] Amadio DM, Adam T, Buletza K. Gender differences in alcohol use and alcohol-related problems: Do they hold for lesbians and gay men? J Gay Lesbian Soc Serv. 2008;20(4):315–27.

[43] Warner J, McKeown E, Griffin M, Johnson K, Ramsay A, Cort C, et al Rates and predictors of mental illness in gay men, lesbians and bisexual men and women: Results from a survey based in England and Wales. The British Journal of Psychiatry. 2004;185(6):479–85. 15572738 10.1192/bjp.185.6.479

[44] Cochran SD, Mays VM. Relation between psychiatric syndromes and behaviorally defined sexual orientation in a sample of the US population. Am J Epidemiol. 2000;151(5):516–23. 10.1093/oxfordjournals.aje.a010238 10707921 PMC3698226

[45] Wilsnack SC, Hughes TL, Johnson TP, Bostwick WB, Szalacha LA, Benson P, et al Drinking and drinking-related problems among heterosexual and sexual minority women. J Stud Alcohol Drugs. 2008;69(1):129–39. 10.15288/jsad.2008.69.129 18080073

[46] Cochran SD, Keenan C, Schober C, Mays VM. Estimates of alcohol use and clinical treatment needs among homosexually active men and women in the US population. J Consult Clin Psychol. 2000;68(6):1062 10.1037//0022-006x.68.6.1062 11142540 PMC4197972

[47] Sandfort TGM, de Graaf R, Bijl RV, Schnabel P. Same-sex sexual behavior and psychiatric disorders: Findings from the Netherlands Mental Health Survey and Incidence Study (NEMESIS). Arch Gen Psychiatry. 2001;58(1):85–91. 10.1001/archpsyc.58.1.85 11146762

[48] Sandfort TGM, de Graaf R, ten Have M, Ransome Y, Schnabel P. Same-sex sexuality and psychiatric disorders in the second Netherlands Mental Health Survey and Incidence Study (NEMESIS-2). LGBT Health. 2014;1(4):292–301. 10.1089/lgbt.2014.0031 26609539 PMC4655175

[49] Balsam KF, Lehavot K, Beadnell B. Sexual revictimization and mental health: A comparison of lesbians, gay men, and heterosexual women. J Interpers Violence. 2011;26(9):1798–814. 10.1177/0886260510372946 20724297

[50] Jaffe C, Clance PR, Nichols MF, Emshoff JG. The prevalence of alcoholism and feelings of alienation in lesbian and heterosexual women. Journal of Gay & Lesbian Psychotherapy. 2000;3(3–4):25–35.

[51] Brown R, McNair R, Szalacha L, Livingston PM, Hughes T. Cancer risk factors, diagnosis and sexual identity in the Australian Longitudinal Study of Women's Health. Women's Health Issues. 2015;25(5):509–16. 10.1016/j.whi.2015.04.001 26044972

[52] Lhomond B, Saurel-Cubizolles M-J, Michaels S, Group CSF. A multidimensional measure of sexual orientation, use of psychoactive substances, and depression: Results of a national survey on sexual behavior in France. Arch Sex Behav. 2014;43(3):607–19. 10.1007/s10508-013-0124-y 23743831

[53] Hughes TL, Wilsnack SC, Kristjanson AF. Substance use and related problems among us women who identify as mostly heterosexual. BMC Public Health. 2015;15(1):803. 26289792 10.1186/s12889-015-2143-1 PMC4546044

[54] Bauer GR, Jairam JA, Baidoobonso SM. Sexual health, risk behaviors, and substance use in heterosexual-identified women with female sex partners: 2002 US National Survey of Family Growth. Sex Transm Dis. 2010;37(9):531–7. 10.1097/OLQ.0b013e3181d785f4 20502395

[55] Talley AE, Aranda F, Hughes TL, Everett B, Johnson TP. Longitudinal associations among discordant sexual orientation dimensions and hazardous drinking in a cohort of sexual minority women. J Health Soc Behav. 2015;56(2):225–45. 10.1177/0022146515582099 25911224 PMC4456672

[56] Gattis MN, Sacco P, Cunningham-Williams R. Substance use and mental health disorders among heterosexual identified men and women who have same-sex partners or same-sex attraction: Results from the National Epidemiological Survey on Alcohol and Related Conditions. Arch Sex Behav. 2012;41(5):1185–97. 10.1007/s10508-012-9910-1 22549338 PMC4731090

[57] Bostwick WB, Hughes TL, Everett B. Health behavior, status, and outcomes among a community-based sample of lesbian and bisexual women. LGBT Health. 2015;2(2):121–6. 10.1089/lgbt.2014.0074 26790117 PMC4932780

[58] Cochran SD, Ackerman D, Mays VM, Ross MW. Prevalence of non‐medical drug use and dependence among homosexually active men and women in the US population. Addiction. 2004;99(8):989–98. 10.1111/j.1360-0443.2004.00759.x 15265096 PMC4190042

[59] Kecojevic A, Jun HJ, Reisner SL, Corliss HL. Concurrent polysubstance use in a longitudinal study of us youth: Associations with sexual orientation. Addiction. 2017;112(4):614–24. 10.1111/add.13681 27790758 PMC5339035

[60] Operario D, Gamarel KE, Grin BM, Lee JH, Kahler CW, Marshall BDL, et al Sexual minority health disparities in adult men and women in the United States: National Health and Nutrition Examination Survey, 2001–2010. Am J Public Health. 2015;105(10):e27–e34. 10.2105/AJPH.2015.302762 26270288 PMC4566530

[61] Everett B, Mollborn S. Differences in hypertension by sexual orientation among us young adults. J Community Health. 2013;38(3):588–96. 10.1007/s10900-013-9655-3 23397511 PMC3642207

[62] Farmer GW, Jabson JM, Bucholz KK, Bowen DJ. A population-based study of cardiovascular disease risk in sexual-minority women. Am J Public Health. 2013;103(10):1845–50. 10.2105/AJPH.2013.301258 23948018 PMC3780727

[63] Paquette R, Tanton C, Burns F, Prah P, Shahmanesh M, Field N, et al Illicit drug use and its association with key sexual risk behaviours and outcomes: Findings from Britain’s third National Survey of Sexual Attitudes and Lifestyles (Natsal-3). PLoS One. 2017;12(5):e0177922 10.1371/journal.pone.0177922 28542366 PMC5436851

[64] Bolton S-L, Sareen J. Sexual orientation and its relation to mental disorders and suicide attempts: Findings from a nationally representative sample. The Can J Psychiatry. 2011;56(1):35–43. 10.1177/070674371105600107 21324241

[65] Degenhardt L. Drug use and risk behaviour among regular ecstasy users: Does sexuality make a difference? Cult Health Sex. 2005;7(6):599–614. 10.1080/13691050500349875 16864225

[66] Parsons JT, Kelly BC, Wells BE. Differences in club drug use between heterosexual and lesbian/bisexual females. Addict Behav. 2006;31(12):2344–9. 10.1016/j.addbeh.2006.03.006 16632210 PMC2688448

[67] Corliss HL, Grella CE, Mays VM, Cochran SD. Drug use, drug severity, and help-seeking behaviors of lesbian and bisexual women. J Womens Health. 2006;15(5):556–68. 10.1089/jwh.2006.15.556 PMC4174333 16796483

[68] Jeong YM, Veldhuis CB, Aranda F, Hughes TL. Racial/ethnic differences in unmet needs for mental health and substance use treatment in a community‐based sample of sexual minority women. J Clin Nurs. 2016;25(23–24):3557–69. 10.1111/jocn.13477 27461857 PMC5819990

[69] Talley AE, Grimaldo G, Wilsnack SC, Hughes TL, Kristjanson AF. Childhood victimization, internalizing symptoms, and substance use among women who identify as mostly heterosexual. LGBT Health. 2016;3(4):266–74. 10.1089/lgbt.2015.0073 27269733 PMC4976251

[70] Hughes TL, Wilsnack SC, Szalacha LA, Johnson T, Bostwick WB, Seymour R, et al Age and racial/ethnic differences in drinking and drinking-related problems in a community sample of lesbians. J Stud Alcohol. 2006;67(4):579–90. 10.15288/jsa.2006.67.579 16736078

[71] Parks CA, Hughes TL. Alcohol use and alcohol-related problems in self-identified lesbians: An historical cohort analysis. J Lesbian Studies. 2005;9(3):31–44. 10.1300/J155v09n03_04 17548283

[72] Mereish EH, Bradford JB. Intersecting identities and substance use problems: Sexual orientation, gender, race, and lifetime substance use problems. J Stud Alcohol Drugs. 2014;75(1):179–88. 10.15288/jsad.2014.75.179 24411810 PMC3893631

[73] Balsam KF, Molina Y, Blayney JA, Dillworth T, Zimmerman L, Kaysen D. Racial/ethnic differences in identity and mental health outcomes among young sexual minority women. Cultur Divers Ethnic Minor Psychol. 2015;21(3):380–90. 10.1037/a0038680 25642782 PMC4512644

[74] Lewis RJ, Mason TB, Winstead BA, Gaskins M, Irons LB. Pathways to hazardous drinking among racially and socioeconomically diverse lesbian women: Sexual minority stress, rumination, social isolation, and drinking to cope. Psychol Women Q. 2016;40(4):564–81. 10.1177/0361684316662603 28138208 PMC5270712

[75] MacCarthy S, Mena L, Chan PA, Rose J, Simmons D, Riggins R, et al Sexual network profiles and risk factors for STIs among African-American sexual minorities in Mississippi: A cross-sectional analysis. LGBT Health. 2015;2(3):276–81. 10.1089/lgbt.2014.0019 26788677 PMC4713017

[76] Hughes TL, Matthews AK, Razzano L, Aranda F. Psychological distress in African American lesbian and heterosexual women. J Lesbian Studies. 2003;7(1):51–68. 10.1300/J155v07n01_04 24815714

[77] Cochran SD, Mays VM, Alegria M, Ortega AN, Takeuchi D. Mental health and substance use disorders among Latino and Asian American lesbian, gay, and bisexual adults. J Consult Clin Psychol. 2007;75(5):785 10.1037/0022-006X.75.5.785 17907860 PMC2676845

[78] Matthews A, Li C-C, Aranda F, Torres L, Vargas M, Conrad M. The influence of acculturation on substance use behaviors among Latina sexual minority women: The mediating role of discrimination. Subst Use Misuse. 2014;49(14):1888–98. 10.3109/10826084.2014.913632 24941026

[79] Cerezo A. The impact of discrimination on mental health symptomatology in sexual minority immigrant Latinas. Psychol Sex Orientat Gend Divers. 2016;3(3):283.

[80] Yuan N, Duran B, Walters K, Pearson C, Evans-Campbell T. Alcohol misuse and associations with childhood maltreatment and out-of-home placement among urban two-spirit American Indian and Alaska Native people. Int J Environ Res Public Health. 2014;11(10):10461–79. 10.3390/ijerph111010461 25317980 PMC4210990

[81] Dibble SL, Sato N, Haller E. Asians and Native Hawaiian or other Pacific Islanders midlife lesbians' health: A pilot study. Women Ther. 2007;30(3–4):129–43.

[82] Austin EL, Irwin JA. Age differences in the correlates of problematic alcohol use among southern lesbians. J Stud Alcohol Drugs. 2010;71(2):295–8. 10.15288/jsad.2010.71.295 20230728

[83] Bryan AEB, Kim H-J, Fredriksen-Goldsen KI. Factors associated with high-risk alcohol consumption among LGB older adults: The roles of gender, social support, perceived stress, discrimination, and stigma. Gerontologist. 2017;57(suppl_1):S95–S104. 28087799 10.1093/geront/gnw100 PMC5241750

[84] Boehmer U, Miao X, Linkletter C, Clark MA. Adult health behaviors over the life course by sexual orientation. Am J Public Health. 2012;102(2):292–300. 10.2105/AJPH.2011.300334 22390443 PMC3483982

[85] Herrick A, Kuhns L, Kinsky S, Johnson A, Garofalo R. Demographic, psychosocial, and contextual factors associated with sexual risk behaviors among young sexual minority women. J Am Psychiatr Nurses Assoc. 2013;19(6):345–55. 10.1177/1078390313511328 24217447

[86] Litt DM, Lewis MA, Rhew IC, Hodge KA, Kaysen DL. Reciprocal relationships over time between descriptive norms and alcohol use in young adult sexual minority women. Psychol Addict Behav. 2015;29(4):885 10.1037/adb0000122 26478944 PMC4701630

[87] D'Augelli AR, Grossman AH. Disclosure of sexual orientation, victimization, and mental health among lesbian, gay, and bisexual older adults. J Interpers Violence. 2001;16(10):1008–27.

[88] Fredriksen-Goldsen KI, Kim H-J, Barkan SE, Muraco A, Hoy-Ellis CP. Health disparities among lesbian, gay, and bisexual older adults: Results from a population-based study. Am J Public Health. 2013;103(10):1802–9. 10.2105/AJPH.2012.301110 23763391 PMC3770805

[89] Masini BE, Barrett HA. Social support as a predictor of psychological and physical well-being and lifestyle in lesbian, gay, and bisexual adults aged 50 and over. J Gay Lesbian Soc Serv. 2008;20(1–2):91–110.

[90] Lee JH, Gamarel KE, Bryant KJ, Zaller ND, Operario D. Discrimination, mental health, and substance use disorders among sexual minority populations. LGBT Health. 2016;3(4):258–65. 10.1089/lgbt.2015.0135 27383512 PMC4976222

[91] McCabe SE, Bostwick WB, Hughes TL, West BT, Boyd CJ. The relationship between discrimination and substance use disorders among lesbian, gay, and bisexual adults in the United States. Am J Public Health. 2010;100(10):1946–52. 10.2105/AJPH.2009.163147 20075317 PMC2937001

[92] Frisell T, Lichtenstein P, Rahman Q, Långström N. Psychiatric morbidity associated with same-sex sexual behaviour: Influence of minority stress and familial factors. Psychol Med. 2010;40(2):315–24. 10.1017/S0033291709005996 19460186

[93] Everett BG, Hatzenbuehler ML, Hughes TL. The impact of civil union legislation on minority stress, depression, and hazardous drinking in a diverse sample of sexual-minority women: A quasi-natural experiment. Soc Sci Med. 2016;169:180–90. 10.1016/j.socscimed.2016.09.036 27733300 PMC5364018

[94] Amadio DM. Internalized heterosexism, alcohol use, and alcohol-related problems among lesbians and gay men. Addict Behav. 2006;31(7):1153–62. 10.1016/j.addbeh.2005.08.013 16183207

[95] Lea T, de Wit J, Reynolds R. Minority stress in lesbian, gay, and bisexual young adults in Australia: Associations with psychological distress, suicidality, and substance use. Arch Sex Behav. 2014;43(8):1571–8. 10.1007/s10508-014-0266-6 24573397

[96] Lehavot K, Simoni JM. The impact of minority stress on mental health and substance use among sexual minority women. J Consult Clin Psychol. 2011;79(2):159 10.1037/a0022839 21341888 PMC4059829

[97] Weber G. Using to numb the pain: Substance use and abuse among lesbian, gay, and bisexual individuals. J Ment Health Couns. 2008;30(1):31–48.

[98] Wilson SM, Gilmore AK, Rhew IC, Hodge KA, Kaysen DL. Minority stress is longitudinally associated with alcohol-related problems among sexual minority women. Addict Behav. 2016;61:80–3. 10.1016/j.addbeh.2016.05.017 27249806 PMC4915988

[99] Nawyn SJ, Richman JA, Rospenda KM, Hughes TL. Sexual identity and alcohol-related outcomes: Contributions of workplace harassment. J Subst Abuse. 2000;11(3):289–304. 10.1016/s0899-3289(00)00028-6 11026127

[100] Hequembourg AL, Dearing RL. Exploring shame, guilt, and risky substance use among sexual minority men and women. J Homosex. 2013;60(4):615–38. 10.1080/00918369.2013.760365 23469820 PMC3621125

[101] Hendy HM, Joseph LJ, Can SH. Repressed anger mediates associations between sexual minority stressors and negative psychological outcomes in gay men and lesbian women. J Gay Lesbian Ment Health. 2016;20(3):280–96.

[102] Corte C, Matthews AK, Stein KF, Lee C-K. Early drinking onset moderates the effect of sexual minority stress on drinking identity and alcohol use in sexual and gender minority women. Psychol Sex Orientat Gend Divers. 2016;3(4):480–8.

[103] Boyle SC, LaBrie JW, Costine LD, Witkovic YD. “It's how we deal”: Perceptions of LGB peers' use of alcohol and other drugs to cope and sexual minority adults' own coping motivated substance use following the Pulse nightclub shooting. Addict Behav. 2017;65:51–5. 10.1016/j.addbeh.2016.10.001 27728830 PMC5140745

[104] Feinstein BA, Dyar C, London B. Are outness and community involvement risk or protective factors for alcohol and drug abuse among sexual minority women? Arch Sex Behav. 2017;46(5):1411–23. 10.1007/s10508-016-0790-7 27473072

[105] Saphira M, Glover M. The effects of coming out on relationships and health. J Lesbian Studies. 2001;5(1–2):183–94. 10.1300/J155v05n01_12 24807574

[106] Rothman EF, Sullivan M, Keyes S, Boehmer U. Parents' supportive reactions to sexual orientation disclosure associated with better health: Results from a population-based survey of LGB adults in Massachusetts. J Homosex. 2012;59(2):186–200. 10.1080/00918369.2012.648878 22335417 PMC3313451

[107] Nguyen TQ, Bandeen-Roche K, German D, Nguyen NTT, Bass JK, Knowlton AR. Negative treatment by family as a predictor of depressive symptoms, life satisfaction, suicidality, and tobacco/alcohol use in Vietnamese sexual minority women. LGBT Health. 2016;3(5):357–65. 10.1089/lgbt.2015.0017 27219025 PMC5073240

[108] Drabble L, Trocki KF, Hughes TL, Korcha RA, Lown AE. Sexual orientation differences in the relationship between victimization and hazardous drinking among women in the National Alcohol Survey. Psychol Addict Behav. 2013;27(3):639 10.1037/a0031486 23438246 PMC3823232

[109] Reisner SL, Falb KL, Wagenen AV, Grasso C, Bradford J. Sexual orientation disparities in substance misuse: The role of childhood abuse and intimate partner violence among patients in care at an urban community health center. Subst Use Misuse. 2013;48(3):274–89. 10.3109/10826084.2012.755702 23368669 PMC3918899

[110] Mereish EH, O’Cleirigh C, Bradford JB. Interrelationships between LGBT-based victimization, suicide, and substance use problems in a diverse sample of sexual and gender minorities. Psychol Health Med. 2014;19(1):1–13. 10.1080/13548506.2013.780129 23535038 PMC3809157

[111] Valentine SE, Elsesser S, Grasso C, Safren SA, Bradford JB, Mereish E, et al The predictive syndemic effect of multiple psychosocial problems on health care costs and utilization among sexual minority women. J Urban Health. 2015;92(6):1092–104. 10.1007/s11524-015-9989-5 26438415 PMC4675741

[112] Bimbi DS, Palmadessa NA, Parsons JT. Substance use and domestic violence among urban gays, lesbians and bisexuals. J LGBT Health Res. 2007;3(2):1–7. 10.1300/J463v03n02_01 19835036

[113] Kelly BC, Izienicki H, Bimbi DS, Parsons JT. The intersection of mutual partner violence and substance use among urban gays, lesbians, and bisexuals. Deviant Behavior. 2011;32(5):379–404.

[114] Finlinson HA, Robles RR, Colón HM, Lopez MS, del Carmen Negrdn M, Oliver‐Vélez D, et al Puerto Rican drug users’ experiences of physical and sexual abuse: Comparisons based on sexual identities. J Sex Res. 2003;40(3):277–85. 10.1080/00224490309552192 14533022

[115] Eaton L, Kaufman M, Fuhrel A, Cain D, Cherry C, Pope H, et al Examining factors co-existing with interpersonal violence in lesbian relationships. J Fam Violence. 2008;23(8):697–705.

[116] Otis MD, Oser CB, Staton-Tindall M. Violent victimization and substance dependency: Comparing rural incarcerated heterosexual and sexual minority women. J Soc Work Pract Addict. 2016;16(1–2):176–201. 10.1080/1533256X.2016.1143372 27660590 PMC5027961

[117] Mattocks KM, Sadler A, Yano EM, Krebs EE, Zephyrin L, Brandt C, et al Sexual victimization, health status, and VA healthcare utilization among lesbian and bisexual OEF/OIF veterans. J Gen Intern Med. 2013;28(2):604–8. 10.1007/s11606-013-2357-9 PMC3695265 23807072

[118] Hughes TL, Johnson T, Wilsnack SC. Sexual assault and alcohol abuse: A comparison of lesbians and heterosexual women. J Subst Abuse. 2001;13(4):515–32. 10.1016/s0899-3289(01)00095-5 11775080

[119] Hughes TL, Johnson TP, Wilsnack SC, Szalacha LA. Childhood risk factors for alcohol abuse and psychological distress among adult lesbians. Child Abuse Negl. 2007;31(7):769–89. 10.1016/j.chiabu.2006.12.014 17628667 PMC2600503

[120] Hughes TL, Szalacha LA, Johnson TP, Kinnison KE, Wilsnack SC, Cho Y. Sexual victimization and hazardous drinking among heterosexual and sexual minority women. Addict Behav. 2010;35(12):1152–6. 10.1016/j.addbeh.2010.07.004 20692771 PMC3006188

[121] Hughes TL, Johnson TP, Steffen AD, Wilsnack SC, Everett B. Lifetime victimization, hazardous drinking, and depression among heterosexual and sexual minority women. LGBT Health. 2014;1(3):192–203. 10.1089/lgbt.2014.0014 26789712 PMC5089701

[122] Mason TB, Lewis RJ, Gargurevich M, Kelley ML. Minority stress and intimate partner violence perpetration among lesbians: Negative affect, hazardous drinking, and intrusiveness as mediators. Psychol Sex Orientat Gend Divers. 2016;3(2):236.

[123] Lewis RJ, Padilla MA, Milletich RJ, Kelley ML, Winstead BA, Lau-Barraco C, et al Emotional distress, alcohol use, and bidirectional partner violence among lesbian women. Violence Against Women. 2015;21(8):917–38. 10.1177/1077801215589375 26062874 PMC4490938

[124] Kelley ML, Lewis RJ, Mason TB. Discrepant alcohol use, intimate partner violence, and relationship adjustment among lesbian women and their same-sex intimate partners. J Fam Violence. 2015;30(8):977–86. 10.1007/s10896-015-9743-5 26478657 PMC4607288

[125] Sandfort T, Frazer MS, Matebeni Z, Reddy V, Southey-Swartz I. Histories of forced sex and health outcomes among Southern African lesbian and bisexual women: A cross-sectional study. BMC Womens Health. 2015;15(22). 10.1186/s12905-015-0181-6 PMC4359450 25783653

[126] Branstrom R. Minority stress factors as mediators of sexual orientation disparities in mental health treatment: A longitudinal population-based study. J Epidemiol Community Health. 2017;71(5):446–52. 10.1136/jech-2016-207943 28043996 PMC5484026

[127] Hequembourg AL, Livingston JA, Parks KA. Sexual victimization and associated risks among lesbian and bisexual women. Violence Against Women. 2013;19(5):634–57. 10.1177/1077801213490557 23759663 PMC3706505

[128] Molina Y, Marquez JH, Logan DE, Leeson CJ, Balsam KF, Kaysen DL. Current intimate relationship status, depression, and alcohol use among bisexual women: The mediating roles of bisexual-specific minority stressors. Sex Roles. 2015;73(1–2):43–57. 26456995 10.1007/s11199-015-0483-z PMC4594946

[129] Reczek C, Liu H, Spiker R. A population‐based study of alcohol use in same‐sex and different‐sex unions. J Marriage Fam. 2014;76(3):557–72. 10.1111/jomf.12113 24860195 PMC4029769

[130] Drabble L, Trocki KF, Klinger JL. Religiosity as a protective factor for hazardous drinking and drug use among sexual minority and heterosexual women: Findings from the National Alcohol Survey. Drug Alcohol Depend. 2016;161:127–34. 10.1016/j.drugalcdep.2016.01.022 26857897 PMC4792700

[131] Hughes TL. Lesbians’ drinking patterns: Beyond the data. Subst Use Misuse. 2003;38(11–13):1739–58. 10.1081/ja-120024239 14582576

[132] Trocki KF, Drabble L, Midanik L. Use of heavier drinking contexts among heterosexuals, homosexuals and bisexuals: Results from a national household probability survey. J Stud Alcohol. 2005;66(1):105–10. 10.15288/jsa.2005.66.105 15830910

[133] Parks CA, Heller NR. The influence of early drinking contexts on current drinking among adult lesbian and bisexual women. J Am Psychiatr Nurses Assoc. 2013;19(5):241–54. 10.1177/1078390313500145 23942089

[134] Parks CA, Hughes TL, Kinnison KE. The relationship between early drinking contexts of women “coming out” as lesbian and current alcohol use. J LGBT Health Res. 2007;3(3):73–90. 10.1080/15574090802095823 19042906

[135] Trocki K, Drabble L. Bar patronage and motivational predictors of drinking in the San Francisco Bay Area: Gender and sexual identity differences. J Psychoactive Drugs. 2008;40(sup5):345–56. 10.1080/02791072.2008.10400662 PMC2801063 19248392

[136] Hyde Z, Comfort J, McManus A, Brown G, Howat P. Alcohol, tobacco and illicit drug use amongst same-sex attracted women: Results from the western Australian lesbian and bisexual women's health and well-being survey. BMC Public Health. 2009;9(1):317. 19725956 10.1186/1471-2458-9-317 PMC2749041

[137] Boyle SC, LaBrie JW, Witkovic YD. Do lesbians overestimate alcohol use norms? Exploring the potential utility of personalized normative feedback interventions to reduce high-risk drinking in Southern California lesbian communities. J Gay Lesbian Soc Serv. 2016;28(3):179–94. 10.1080/10538720.2016.1190677 28579731 PMC5451126

[138] Johnson TP, Hughes TL, Cho YI, Wilsnack SC, Aranda F, Szalacha LA. Hazardous drinking, depression, and anxiety among sexual-minority women: Self-medication or impaired functioning? J Stud Alcohol Drugs. 2013;74(4):565–75. 10.15288/jsad.2013.74.565 23739020 PMC3711347

[139] Mereish EH, Lee JH, Gamarel KE, Zaller ND, Operario D. Sexual orientation disparities in psychiatric and drug use disorders among a nationally representative sample of women with alcohol use disorders. Addict Behav. 2015;47:80–5. 10.1016/j.addbeh.2015.03.023 25899096 PMC4417371

[140] Span SA, Derby PL. Depressive symptoms moderate the relation between internalized homophobia and drinking habits. J Gay Lesbian Soc Serv. 2009;21(1):1–12.

[141] Talley AE, Tomko RL, Littlefield AK, Trull TJ, Sher KJ. The influence of general identity disturbance on reports of lifetime substance use disorders and related outcomes among sexual minority adults with a history of substance use. Psychol Addict Behav. 2011;25(3):530–41. 10.1037/a0023022 21480677 PMC4106243

[142] McBee-Strayer SM, Rogers JR. Lesbian, gay, and bisexual suicidal behavior: Testing a constructivist model. Suicide Life Threat Behav. 2002;32(3):272–83. 10.1521/suli.32.3.272.22171 12374473

[143] Moller CI, Tait RJ, Byrne DG. Self‐harm, substance use and psychological distress in the Australian general population. Addiction. 2013;108(1):211–20. 10.1111/j.1360-0443.2012.04021.x 22788830

[144] Husky MM, Guignard R, Beck F, Michel G. Risk behaviors, suicidal ideation and suicide attempts in a nationally representative French sample. J Affect Disord. 2013;151(3):1059–65. 10.1016/j.jad.2013.08.035 24070905

[145] Hatzenbuehler ML, Slopen N, McLaughlin KA. Stressful life events, sexual orientation, and cardiometabolic risk among young adults in the United States. Health Psychol. 2014;33(10):1185 10.1037/hea0000126 25133830 PMC4436691

[146] Rosario M, Li F, Wypij D, Roberts AL, Corliss HL, Charlton BM, et al Disparities by sexual orientation in frequent engagement in cancer-related risk behaviors: A 12-year follow-up. Am J Public Health. 2016;106(4):698–706. 10.2105/AJPH.2015.302977 26794176 PMC4785035

[147] Blosnich JR, Farmer GW, Lee JGL, Silenzio VMB, Bowen DJ. Health inequalities among sexual minority adults: Evidence from ten US states, 2010. Am J Prev Med. 2014;46(4):337–49. 10.1016/j.amepre.2013.11.010 24650836 PMC4102129

[148] Gonzales G, Przedworski J, Henning-Smith C. Comparison of health and health risk factors between lesbian, gay, and bisexual adults and heterosexual adults in the United States: Results from the National Health Interview Survey. JAMA Intern Med. 2016;176(9):1344–51. 10.1001/jamainternmed.2016.3432 27367843

[149] Gonzales G, Henning-Smith C. Health disparities by sexual orientation: Results and implications from the behavioral risk factor surveillance system. J Community Health. 2017;42(6):1163–72. 10.1007/s10900-017-0366-z 28466199

[150] Jackson CL, Agénor M, Johnson DA, Austin SB, Kawachi I. Sexual orientation identity disparities in health behaviors, outcomes, and services use among men and women in the United States: A cross-sectional study. BMC Public Health. 2016;16(1):807 10.1186/s12889-016-3467-1 27534616 PMC4989521

[151] Matthews AK, Cho YI, Hughes T, Wilsnack SC, Johnson T, Martin K. The relationships of sexual identity, hazardous drinking, and drinking expectancies with risky sexual behaviors in a community sample of lesbian and bisexual women. J Am Psychiatr Nurses Assoc. 2013;19(5):259–70. 10.1177/1078390313505644 24071822 PMC5088734

[152] Estrich CG, Gratzer B, Hotton AL. Differences in sexual health, risk behaviors, and substance use among women by sexual identity: Chicago, 2009–2011. Sex Transm Dis. 2014;41(3):194–9. 10.1097/OLQ.0000000000000091 24521726

[153] Soto-Salgado M, Colón-López V, M. Perez C, Muñoz C, Marrero E, Suárez E, et al Same-sex behavior and its relationship with sexual and health-related practices among a population-based sample of women in Puerto Rico: Implications for cancer prevention and control. Int J Sex Health. 2016;28(4):296–305. 10.1080/19317611.2016.1223250 28286595 PMC5341788

[154] Adamczyk A, Boyd KA, Hayes BE. Place matters: Contextualizing the roles of religion and race for understanding americans' attitudes about homosexuality. Soc Sci Res. 2016;57:1–16. 10.1016/j.ssresearch.2016.02.001 26973028

[155] Ellison CG, Acevedo GA, Ramos‐Wada AI. Religion and attitudes toward same‐sex marriage among US Latinos. Soc Sci Q. 2011;92(1):35–56. 10.1111/j.1540-6237.2011.00756.x 21523946

[156] Scheitle CP, Wolf JK. Religion and sexual identity fluidity in a national three-wave panel of US adults. Arch Sex Behav. 2018;47(4):1085–94. 10.1007/s10508-017-0979-4 28357526

[157] Sherkat DE, De Vries KM, Creek S. Race, religion, and opposition to same‐sex marriage. Soc Sci Q. 2010;91(1):80–98.

[158] Anderssen N, Malterud K. Oversampling as a methodological strategy for the study of self-reported health among lesbian, gay and bisexual populations. Scand J Public Health. 2017;45(6):637–46. 10.1177/1403494817717407 28675963

[159] Drabble LA, Trocki KF, Korcha RA, Klinger JL, Veldhuis CB, Hughes TL. Comparing substance use and mental health outcomes among sexual minority and heterosexual women in probability and non-probability samples. Drug Alcohol Depend. 2018;185:285–92. 10.1016/j.drugalcdep.2017.12.036 29482053 PMC5889720

[160] Henderson ER, Blosnich JR, Herman JL, Meyer IH. Considerations on sampling in transgender health disparities research. LGBT Health. 2019;6(6):267–70. 10.1089/lgbt.2019.0069 31295043 PMC6740154

[161] Greenfield TK, Kerr WC. Alcohol measurement methodology in epidemiology: Recent advances and opportunities. Addiction. 2008;103(7):1082–99. 10.1111/j.1360-0443.2008.02197.x 18422826 PMC2782942

[162] Bauer GR, Brennan DJ. The problem with ‘behavioral bisexuality': Assessing sexual orientation in survey research. J Bisex. 2013;13(2):148–65.

[163] Midanik LT, Drabble L, Trocki K, Sell RL. Sexual orientation and alcohol use: Identity versus behavior measures. J LGBT Health Res. 2007;3(1):25–35. 10.1300/j463v03n01_04 18029313

[164] McCabe SE, Hughes TL, Bostwick W, Morales M, Boyd CJ. Measurement of sexual identity in surveys: Implications for substance abuse research. Arch Sex Behav. 2012;41(3):649–57. 10.1007/s10508-011-9768-7 21573706 PMC3233651

[165] Everett B. Sexual orientation identity change and depressive symptoms: A longitudinal analysis. J Health Soc Behav. 2015;56(1):37–58. 10.1177/0022146514568349 25690912 PMC4442487

[166] Everett BG, Talley AE, Hughes TL, Wilsnack SC, Johnson TP. Sexual identity mobility and depressive symptoms: A longitudinal analysis of moderating factors among sexual minority women. Arch Sex Behav. 2016;45(7):1731–44. 10.1007/s10508-016-0755-x 27255306 PMC5500256

[167] Ott MQ, Wypij D, Corliss HL, Rosario M, Reisner SL, Gordon AR, et al Repeated changes in reported sexual orientation identity linked to substance use behaviors in youth. J Adolesc Health. 2013;52(4):465–72. 10.1016/j.jadohealth.2012.08.004 23298999 PMC3608814

[168] Bowleg L. The problem with the phrase women and minorities: Intersectionality—an important theoretical framework for public health. Am J Public Health. 2012;102(7):1267–73. 10.2105/AJPH.2012.300750 22594719 PMC3477987

[169] Logie CH, Alaggia R, Rwigema M-J. A social ecological approach to understanding correlates of lifetime sexual assault among sexual minority women in Toronto, Canada: Results from a cross-sectional internet-based survey. Health Educ Res. 2014;29(4):671–82. 10.1093/her/cyt119 24412812 PMC4101185

[170] Fredriksen-Goldsen KI, Simoni JM, Kim H-J, Lehavot K, Walters KL, Yang J, et al The health equity promotion model: Reconceptualization of lesbian, gay, bisexual, and transgender (LGBT) health disparities. Am J Orthopsychiatry. 2014;84(6):653 10.1037/ort0000030 25545433 PMC4350932

[171] Ryan C, Huebner D, Diaz RM, Sanchez J. Family rejection as a predictor of negative health outcomes in white and Latino lesbian, gay, and bisexual young adults. Pediatrics. 2009;123(1):346–52. 10.1542/peds.2007-3524 19117902

[172] Michalak L, Trocki K, Bond J. Religion and alcohol in the US National Alcohol Survey: How important is religion for abstention and drinking? Drug Alcohol Depend. 2007;87(2–3):268–80. 10.1016/j.drugalcdep.2006.07.013 16987610

[173] Drabble L, Veldhuis CB, Riley BB, Rostosky S, Hughes TL. Relationship of religiosity and spirituality to hazardous drinking, drug use, and depression among sexual minority women. J Homosex. 2018;65(13):1734–57. 10.1080/00918369.2017.1383116 28929909 PMC5860995

[174] Bush R, Brown R, McNair R, Orellana L, Lubman DI, Staiger PK. Effectiveness of a culturally tailored sms alcohol intervention for same-sex attracted women: Protocol for an rct. BMC Womens Health. 2019;19(1):29 10.1186/s12905-019-0729-y 30728002 PMC6364437

[175] Allen JL, Mowbray O. Sexual orientation, treatment utilization, and barriers for alcohol related problems: Findings from a nationally representative sample. Drug Alcohol Depend. 2016;161:323–30. 10.1016/j.drugalcdep.2016.02.025 26936411

[176] McCabe SE, West BT, Hughes TL, Boyd CJ. Sexual orientation and substance abuse treatment utilization in the United States: Results from a national survey. J Subst Abuse Treat. 2013;44(1):4–12. 10.1016/j.jsat.2012.01.007 22444421 PMC3388170

[177] Lipsky S, Krupski A, Roy-Byrne P, Huber A, Lucenko BA, Mancuso D. Impact of sexual orientation and co-occurring disorders on chemical dependency treatment outcomes. J Stud Alcohol Drugs. 2012;73(3):401–12. 10.15288/jsad.2012.73.401 22456245

[178] Deren S, Cortes T, Dickson VV, Guilamo-Ramos V, Han BH, Karpiak S, et al Substance use among older people living with HIV: Challenges for health care providers. Front Public Health. 2019;7:94 10.3389/fpubh.2019.00094 31069208 PMC6491638

[179] Sandfort TGM, Knox JR, Alcala C, El-Bassel N, Kuo I, Smith LR. Substance use and HIV risk among men who have sex with men in Africa: A systematic review. J Acquir Immune Defic Syndr. 2017;76(2):e34–e46. 10.1097/QAI.0000000000001462 28903126 PMC5647883

[180] Same-sex marriage: Global comparisons [Internet]. 2019 [cited 2019 August 1]. Available from: https://www.cfr.org/backgrounder/same-sex-marriage-global-comparisons.

[181] Raifman J, Galea S. The new US "conscience and religious freedom division": Imposing religious beliefs on others. Am J Public Health. 2018;108(7):889–90. 10.2105/AJPH.2018.304488 29874507 PMC5993366

[182] Veldhuis CB, Drabble L, Riggle EDB, Wootton AR, Hughes TL. "We won't go back into the closet now without one hell of a fight": Effects of the 2016 presidential election on sexual minority women's and gender minorities' stigma-related concerns. Sex Res Social Policy. 2018;15(1):12–24.

